# Molecular drug susceptibility testing and strain typing of tuberculosis by DNA hybridization

**DOI:** 10.1371/journal.pone.0212064

**Published:** 2019-02-07

**Authors:** Hillary N. Wood, Tom Venken, Hanny Willems, An Jacobs, Ana Júlia Reis, Pedro Eduardo Almeida da Silva, Susanne Homolka, Stefan Niemann, Kyle H. Rohde, Jef Hooyberghs

**Affiliations:** 1 Division of Immunity and Pathogenesis, Burnett School of Biomedical Sciences, College of Medicine, University of Central Florida, Orlando, Florida, United States of America; 2 Flemish Institute for Technological Research, VITO, Mol, Belgium; 3 Laboratory of Tuberculosis, Faculty of Medicine, Universidade Federal do Rio Grande- FURG, Rio Grande so Sul, RS, Brazil; 4 Molecular and Experimental Mycobacteriology, Research Center Borstel, Borstel, Germany; 5 German Center for Infection Research, Borstel, Germany; 6 Theoretical Physics, Hasselt University, Diepenbeek, Belgium; Jamia Hamdard, INDIA

## Abstract

In *Mycobacterium tuberculosis (Mtb*) the detection of single nucleotide polymorphisms (SNPs) is of high importance both for diagnostics, since drug resistance is primarily caused by the acquisition of SNPs in multiple drug targets, and for epidemiological studies in which strain typing is performed by SNP identification. To provide the necessary coverage of clinically relevant resistance profiles and strain types, nucleic acid-based measurement techniques must be able to detect a large number of potential SNPs. Since the *Mtb* problem is pressing in many resource-poor countries, requiring low-cost point-of-care biosensors, this is a non-trivial technological challenge. This paper presents a proof-of-concept in which we chose simple DNA-DNA hybridization as a sensing principle since this can be transferred to existing low-cost hardware platforms, and we pushed the multiplex boundaries of it. With a custom designed probe set and a physicochemical-driven data analysis it was possible to simultaneously detect the presence of SNPs associated with first- and second-line drug resistance and *Mtb* strain typing. We have demonstrated its use for the identification of drug resistance and strain type from a panel of phylogenetically diverse clinical strains. Furthermore, reliable detection of the presence of a minority population (<5%) of drug-resistant *Mtb* was possible.

## Introduction

Tuberculosis (TB), caused by *Mycobacterium tuberculosis* (*Mtb*), remains a major global health problem and is responsible for 10.4 million new infections and 1.8 million deaths annually [1]. The problem is exacerbated by the emergence of drug-resistant MTBC strains. According to the World Health Organization in 2014, 3.3% of new infections and 20% of previously treated cases were multidrug resistant TB (MDR-TB). MDR-TB strains are resistant to the first-line anti-TB drugs rifampin (RIF) and isoniazid (INH). Furthermore, 9.7% of MDR strains were extensively drug resistant (XDR) based on additional resistance to fluoroquinolones (FQ) and an injectable drug (amikacin, capreomycin, or kanamycin) [1]. Non-molecular diagnostic tools for *Mtb* such as sputum smear microscopy (SSM) and culture have inherent limitations that warrant the development of improved tools for the rapid, sensitive, and accurate detection and drug-resistance profiling of *Mtb*. SSM detects <50% of TB cases with even lower detection rates in children and HIV-positive patients and does not provide information on drug resistance [2, 3]. Culture-based testing is time consuming (requiring 2–6 weeks) and is limited by the requirement for elevated biosafety precautions, trained personal, and risk of contamination [4]. To help eradicate this disease it is important therefore to develop a diagnostic tool that can quickly identify *Mtb* and detect resistance to first- and second-line drugs.

Molecular diagnostic tools enable rapid identification, genotypic drug susceptibility testing (DST) and strain typing of bacterial strains. In clinical *Mtb* strains, the main source of drug resistance is the acquisition of single nucleotide polymorphisms (SNPs) that prevent the interaction of drugs with their altered protein target, alter pro-drug activation, or cause upregulation of drug targets [5]. In addition to the detection of drug resistance, SNPs are used in molecular epidemiology studies for strain typing and are considered the most valid markers for phylogenetic classification of clinical strains of the *Mycobacterium tuberculosis* complex (MTBC) [6, 7]. Defined sets of SNPs for differentiating species and strains within the MTBC have been published [8–11]. Several molecular diagnostic tools such as Cepheid’s GeneXpert [12, 13], line probe assays (LiPA) [14] and whole genome sequencing (WGS) technologies are available, but the need for high multiplexing with a simple low-cost robust solution for resource-poor countries is currently still unmet.

**Fig 1:**
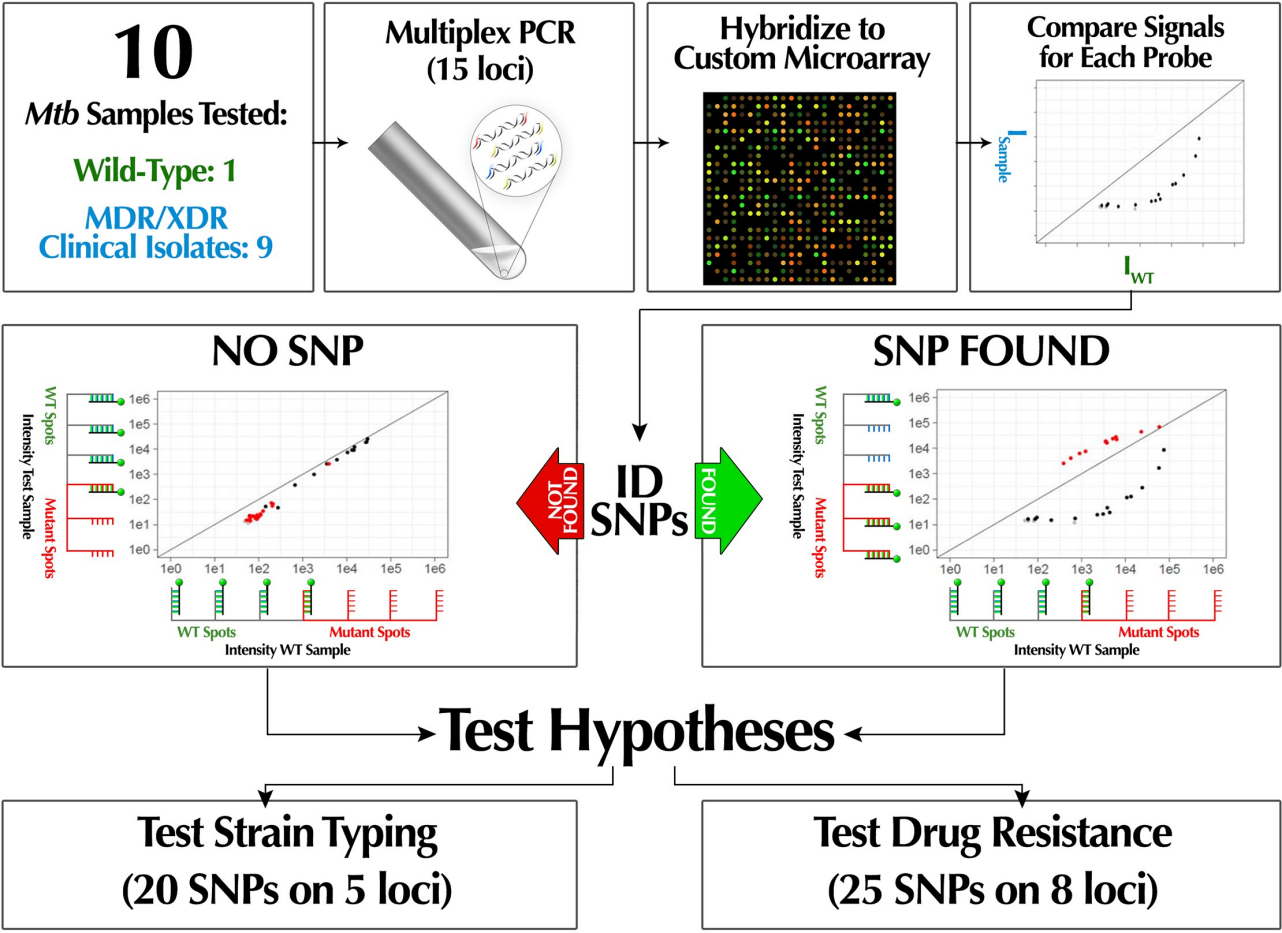
Scheme depicting experimental workflow and data interpretation. A 15-loci multiplex PCR developed in this study serves to generate sufficient analyte DNA from the targeted genes relevant to drug resistance and strain typing. Conceptual diagrams overlayed onto example dot plots of microarray data illustrate how hybridization signals from each probe set can distinguish between two alleles differing by a single polymorphism. Comparison with results from wild-type control samples and alternative mutant alleles allows determination of the specific nucleotide at the tested position.

The goal of this project was to develop a proof-of-concept, multi-purpose DNA hybridization-based method capable of MTBC detection, genomic DST, and strain typing through the detection of SNPs. We designed a 15-loci multiplex PCR, followed by amplicon detection with a custom, high-density microarray (**Fig 1**) targeting the most common mutations associated with first- and second-line drug resistance and the strain typing SNPs established by Homolka *et al*. [8]. In principle any hybridization-based hardware platform could have been used for this study, but in the current proof-of-principle stage the maturity of the microarray technology was an advantage. What is crucial about the presented method is that it relies on an established thermodynamic framework [15–19] using unique probe sets which contain mismatch mutations to improve the dynamic range of the detection. These probe sets enable the specific identification of targeted mutations even when present at ~1% relative to wild-type.

## Materials and methods

### Mycobacterial strains used in this study

DNA for multiplex PCR was isolated from drug-susceptible wild-type (WT) *Mtb* strain CDC1551 (BEI Resources, ATCC) or clinical isolates containing a variety of SNPs. Clinical isolates used in this study (**Table C in S1 File**) were obtained from archived stocks at the Mycobacteria Laboratory of the Federal University of Rio Grande Brazil or the National Reference Center for Mycobacteria, Borstel, Germany. DNA was extracted using the standardized CTAB (cetyltrimethylammonium bromide-NaCl) method and stored at -20°C [20].

### Multiplex PCR (mPCR)

The multiplex PCR assay was designed to produce 15 amplicons (230–1018 base pairs) from genes associated with detection of *Mtb* (16S rRNA and 23S rRNA), drug resistance (*rpoB*, *katG*, *inhA*, *gyrA*, *embB*, *pncA*, *rpsL*, and *rrs)*, and lineage specific polymorphisms (*Rv0129c*, *Rv1009*, *Rv1811*, *Rv2926*, *and Rv0557)* [8] (**Table A in S1 File)**. The most common mutations associated with drug resistance were identified using the tuberculosis drug resistance mutation database (https://tbdreamdb.ki.se/Info/). The SNPs associated with strain typing were defined by Homolka *et al*. [8]. Nucleotide sequences of *Mtb* CDC1551 were used to design primers for amplicons encompassing the polymorphisms associated with drug resistance and strain typing. Reverse primers contained a 5’ phosphate to allow for digestion of the antisense strand by lambda exonuclease and an adapter sequence (5’-Cy3-aaaaactggcgtcatagctgtttcctgtgtga-3’) for hybridization of microarray probes to mPCR products. mPCR samples contained 3 units of Phusion DNA Polymerase (NEB), 147 nM– 1.176 μM of each primer (**Table A in S1 File**), 200 μM of each NTP, 1x Phusion HF Buffer, 20 ng of *Mtb* DNA, and water to a final reaction volume of 25 μL. The samples were PCR amplified using the following cycling conditions: initial denaturation 98°C for 30 sec, followed by 50 cycles of denaturation at 98°C for 10 sec, annealing at 65°C for 40 sec, extension at 72°C for 1 min, and final extension at 72°C for 2 min. Following mPCR, samples were purified using QIAgen PCR purification kit following manufacturer protocol. The dsDNA samples were then lyophilized before shipping to VITO.

### Gel validation & densitometry

Multiplex PCR samples were run on a 2% agarose gel at 120 V, 10°C for 12.75 hr followed by staining in GelRed (Biotium, Inc., Fremont, CA) for 2 hr. Densitometry was completed using UN-SCAN-IT gel version 7.1 for relative quantification of each product in the multiplex.

### Microarray experimental design

The microarray was designed to enable the specific detection of SNPs associated with *Mtb* drug resistance and strain type. Although amplicons for *16S* and *23S* rRNA were included in the mPCR, we did not include probe sets for these loci on this prototype array because definitive identification of *Mtb* involves distinguishing multiple mutations in hypervariable regions rather than SNPs [21]. Although we envision this as a second-tier assay to provide comprehensive DST and strain typing information on *Mtb* samples detected by existing methods, we could enable species-specific detection of *Mtb* by adding probe sets targeting MTBC specific regions of *16S/23S* rRNA in subsequent versions. Probe sets were designed according to parameters previously established by the Hooyberghs laboratory [16, 18] to enable accurate identification of SNPs. Each probe set is designed to test a specific hypothesis—the presence or absence of a SNP at a defined nucleotide (nt) position. More information on probe set design can be found in the **Supplemental Information (Table B in S1 File)**.

### Microarray experiments

Lyophilized mPCR products were resuspended in elution buffer from the QIAgen PCR purification kit. Next, 2 μg of purified mPCR products were treated with 10 U lambda exonuclease (Fermentas, St.Leon-Rot, Germany) in 1 μL reaction buffer and 7 μL water for 30 minutes at 37°C and 10 minutes at 80°C. The concentration of the resulting ssDNA was measured on a NanoDrop spectrophotometer. For microarray hybridization measurements we used the commercially available Agilent microarray platform and followed a standard protocol with Agilent products. Hybridization mixtures were prepared by combining a Cy3-labeled adapter (5’-Cy3-aaaaactggcgtcatagctgtttcctgtgtga-3’) diluted in nuclease-free water to a final concentration of 0.05 μM with 500 ng ssDNA, 5 μl 10x blocking agent and 25 μl 2x GEx hybridization buffer HI-RPM. Each hybridization mixture (40 μl) containing ssDNA mPCR products of a single test sample was centrifuged at 13,000 rpm for 1 min and then added to one well on the 8×15K custom Agilent slide. Hybridization occurred in an Agilent oven at 65°C for 17 h with rotor setting 10 and the washing was performed according to the manufacturer's instructions. The slides were scanned on an Agilent scanner (G2565BA) at 5-μm resolution and further processed using Agilent Feature Extraction Software (GE1 v5 95 Feb07) that performs automatic gridding, fluorescence intensity measurement, background subtraction and quality checks.

### Analysis of mixed-strain multiplex PCR samples using microarray

To determine the ability of a custom microarray to identify a mixed infection in which mutations are often present in low abundance compared to WT, we simulated this scenario using our mPCR assay to amplify DNA from two strains: *Mtb* CDC1551 (WT) and the MDR-TB clinical isolate 08–1074 (MT) (refer to **Table C in S1 File**). We completed a mPCR with 4 concentrations of MT DNA (1 ng, 250 pg, 62.5 pg, and 15.6 pg) in the presence of excess WT DNA (20 ng) which is equivalent to approximately 5%, 1.25%, 0.31% and 0.08% MT DNA, respectively. The samples were then processed and applied to the microarray as described above.

### Data analysis

**Fig 2:**
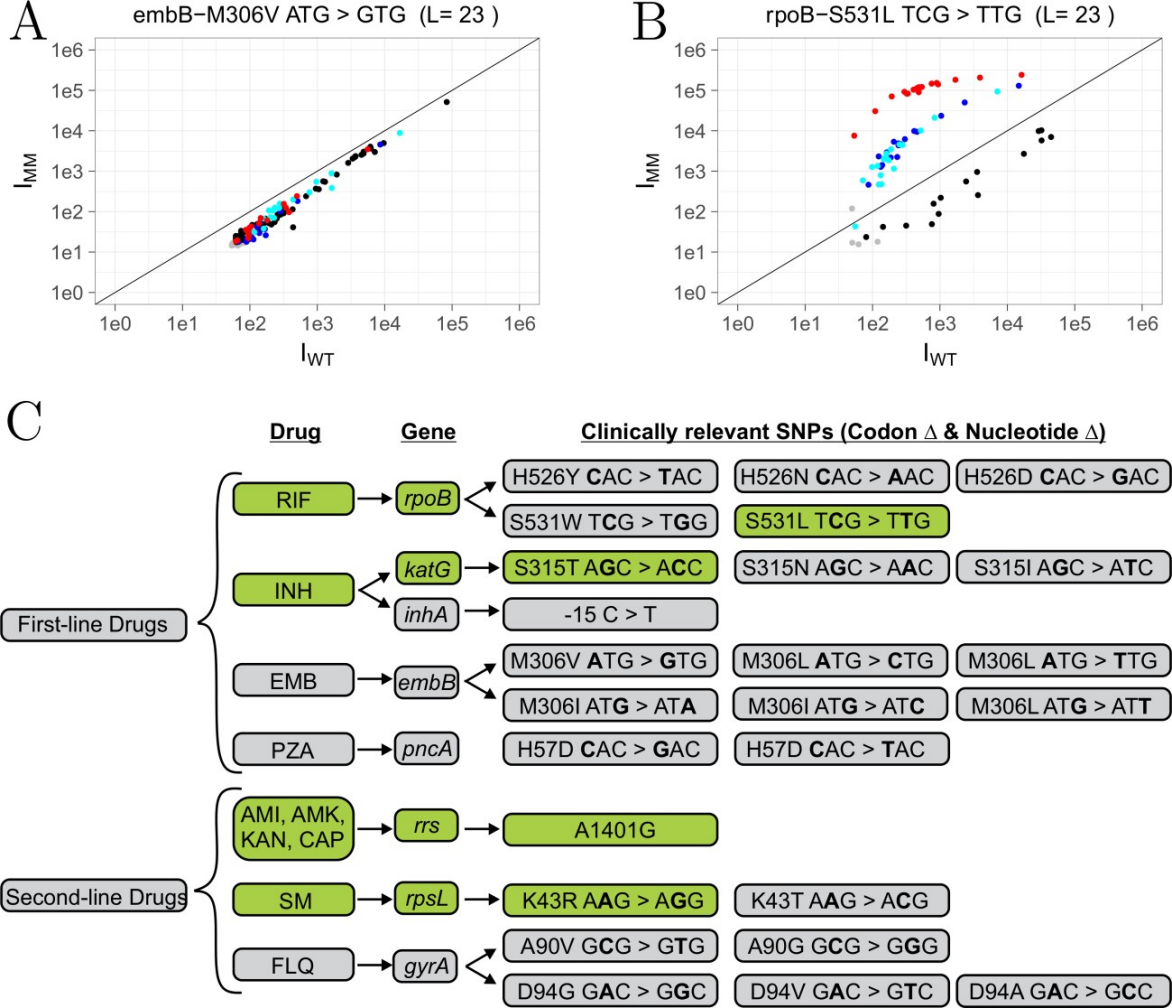
Microarray drug resistance results of XDR-TB strain (08–1186). **A)** Example of wild-type microarray result. **B)** Example of microarray result showing detection of mutation in *rpoB* codon 531. Fluorescence intensities of the wild-type sample (x-axis, I_WT_) are plotted versus the test sample (y-axis, I_MM_, 08–1186). **C)** Drug resistance scheme with detected mutations in green. L, probe length (nt).

**Fig 3:**
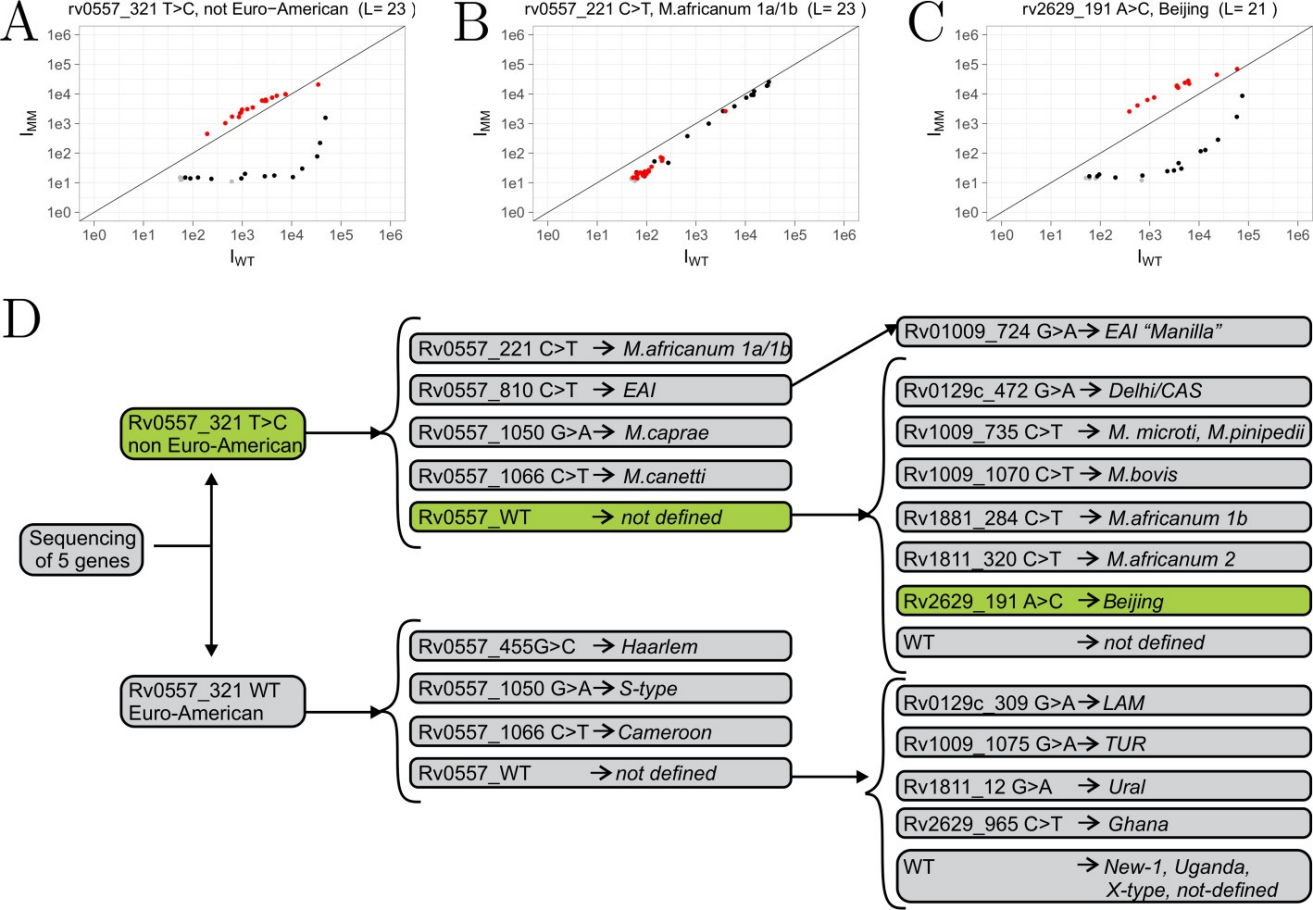
Microarray strain typing results of Beijing strain (08–1186). Fluorescence intensities of the wild-type sample (x-axis, I_WT_) plotted versus the test sample (y-axis, I_MM_). **A)** Microarray result for probe set targeting *Rv0557* nt 321. **B)** Example where no mutation was found in *Rv0557* at nt 221.**C)** Example for *Rv2629* nt 191 A>C confirming Beijing strain type. **D)** Strain typing scheme with detected mutations in green. L, probe length (nt).

A detailed description of the statistical analysis used in this study is described in **Supplemental Information** (**Figure B in S1 File**). The data was analyzed by comparing the fluorescence intensities of the WT reference sample (x-axis) versus the test sample (y-axis) on a scatterplot. The scatterplots allow for easy visualization of the presence or absence of mutation, but results are only considered statistically significant if the data passes three statistical tests. As described by Willems et al. [22], a scatterplot contains branches representing hybridization based on at least two probe sets: the reference branch (or WT, black dots) and the mutant branch (the targeted SNP, red dots). In the case of drug resistance, we tested for all possible SNPs; therefore, two additional branches are formed and are referred to as side branches (alternative SNPs, blue dots). Dots below the detection limit were colored grey in the scatterplots. When the test sample is WT all branches will be on the diagonal line. Alternatively, when the test sample contains a SNP the branches separate; the mutant branch (corresponding to the targeted SNP) will have the highest intensity, the reference branch will have the lowest, and the side branches are intermediate. Furthermore, we designed schemes (**Figs 2C and 3D)** to summarize the results for drug resistance and strain typing. Only clinically relevant mutations are included in the drug resistance scheme. To validate genotypes determined by microarray for all strains, results were compared with whole genome sequencing data where available (strains 03/8864, 03/9532, 03/4850) or targeting sequencing (Eurofins) of PCR amplicons of individual loci from the remaining strains.

## Results

### Design and validation of 15 loci multiplex PCR (mPCR)

The mPCR was designed to amplify fragments of 15 genes (**Table A in S1 File**) associated with the specific detection of *Mtb*, genotypic DST and strain typing of *Mtb*. After optimization of the cycling conditions and primer concentrations (**Table A in S1 File**), we achieved <4-fold variation in relative concentration of PCR products as determined by gel quantification software. Our mPCR assay reliably amplified all 15 specific products at the correct size and with few nonspecific products as shown in **Figure A in S1 File**. Nonspecific products (**Figure A in S1 File**, white arrows) were present in the mPCR, but did not interfere with the accurate and specific detection of 45 mutations (MT).

### Microarray results

#### Genotypic DST

To evaluate the utility of the customized microarray to detect MDR and XDR-TB, we analyzed DNA from 9 clinical isolates with diverse phenotypic and genotypic profiles. Complete results are summarized in **Table C in S1 File** and supplementary figures. Selected results are presented here to illustrate the data analysis process. **Fig 2** shows the drug-resistance SNP profiling results for an XDR-TB strain (08–1186). The scatterplot shown in **Fig 2A** illustrates the pattern observed when a strain is wild-type at codon 306 of *embB* where an **A**TG>**G**TG SNP would indicate ethambutol resistance. In this case, the fluorescence intensity of both wild-type reference (I_WT_) and the test sample signal (I_MM_) lie on a diagonal line indicating that no mutation was found. In contrast, **Fig 2B** demonstrates the presence of a mutation in *rpoB* codon 531 (T**C**G > T**T**G), which is responsible for rifampicin resistance. The most deviating side branch (**Fig 2B,** red dots), the mutant branch, corresponds to the probes complementary to the targeted *rpoB* SNP and therefore has the highest signal intensity compared to WT. Analysis of scatterplots for each targeted mutation (25 SNPs in total) was completed as described in Materials and Methods and the resulting scheme is shown in **Fig 2C**. In all cases, sequence analysis confirmed the genotype assigned based on microarray results (**Table C in S1 File**).

#### Strain typing

To classify *Mtb* isolates based on phylogenetic lineage we designed probe sets based on SNPs in five genes described by Homolka *et al*. as shown in **Fig 3D** (scheme, adopted from Homolka *et al*.[8]). The 19 SNPs tested in each sample were previously shown to be superior to standard MIRU-VNTR typing for defining deep phylogenetics groups with high confidence [8]. Here we demonstrate the strain typing results of strain 08–1186 of the Beijing genotype. **Fig 3A–3C** illustrates the scatterplots of each green box highlighted in **Fig 3D**. The microarray result confirms the presence of two strain-specific SNPs, a non Euro-American SNP (*Rv0557*, nt 321, T>C**—Fig 3A**) and Beijing SNP (*Rv2629*, nt 191, A>C–**Fig 3C**). No other mutations were found as indicated by grey boxes in the scheme. **Fig 3B** shows that no mutation was present in the *Rv0557* gene at position nt 221 as an illustration of a negative result. The strain types for all other strains are summarized in **Table B in S1 File** and corresponding schemes and scatterplots are found in **Supplemental Information (S1 File, pg 12–28).** All strain types were confirmed by sequencing.

#### Probe length comparison

**Fig 4:**
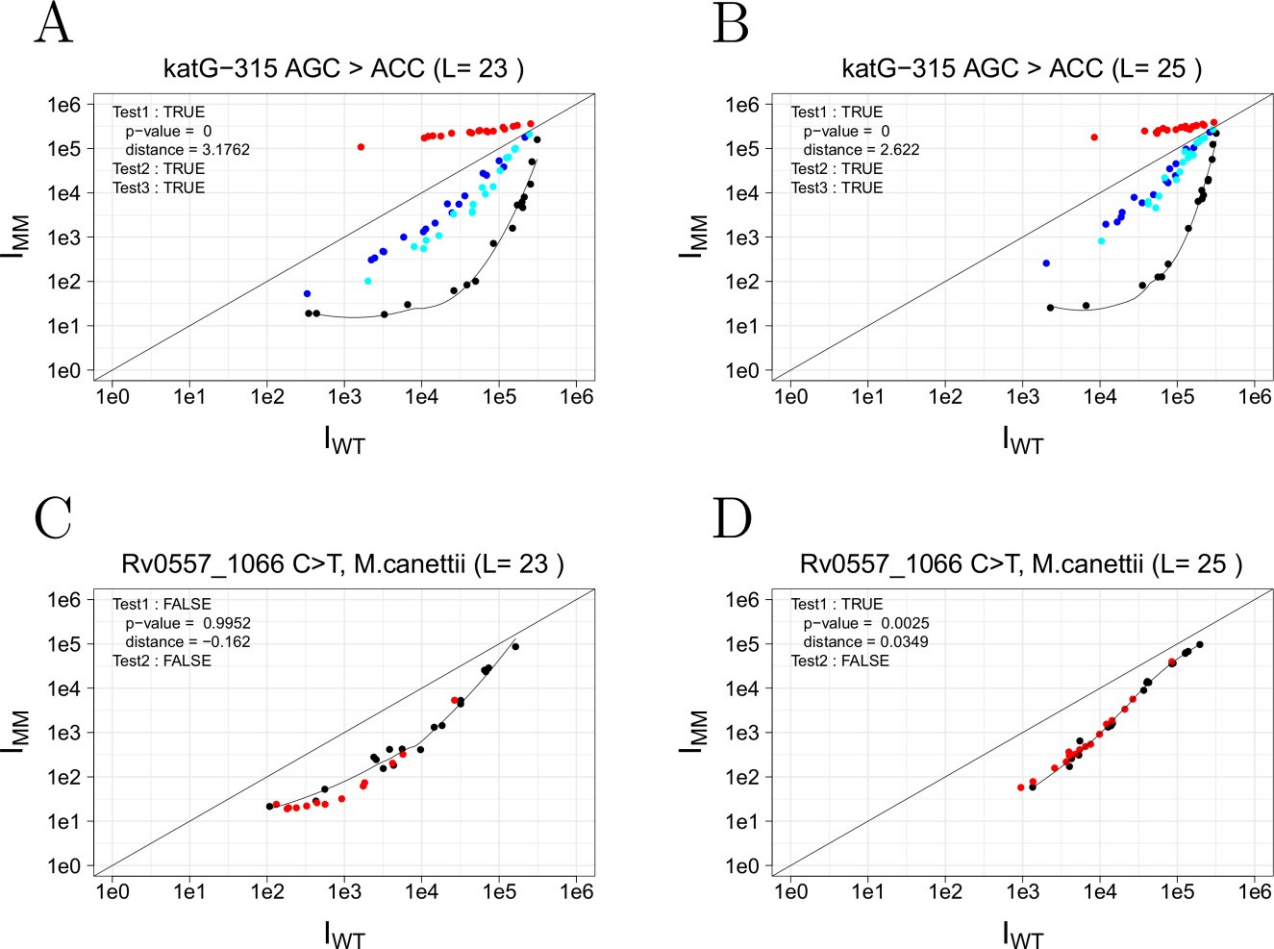
Probe length comparison of strain 08–1186. Fluorescence intensities of the wild-type sample (x-axis, I_WT_) plotted versus the test sample (y-axis, I_MM_). Probe set targeting the SNP (AGC >ACC) in *katG* codon 315 using a probe length (L) of **A)** 23 nt or **B)** 25 nt. Probe set targeting C >T SNP in *Rv0557* at position 1066 using **C)** 23 nt or **D)** 25 nt probe length.

In this proof-of-concept study, each hypothesis (presence/absence of a SNP at a defined location) was tested using multiple probe lengths (between 21–25 nt) to optimize both the microarray signal sensitivity and dynamic range. In some cases, for example the detection of A**G**C > A**C**C in *katG* codon 315 (**Fig 4A and 4B),** the dynamic range was altered when using short (23nt) vs long (25nt) probes without affecting SNP identification. The smaller dynamic range observed with probe length of 25 nt arises from a stronger increase in hybridization free energies between the longer probes and the WT sample compared to the MT sample (**Fig 4B**). However, in a few cases (<1%) probe length affected the ability to confidently determine the presence or absence of a SNP. One of these scenarios is illustrated in **Fig 4C** and **4D**. In this example, the test sample (strain 08–1186, Beijing genotype) does not contain the *M*. *canetti* specific SNP **(**C > T) at position 1066 in *Rv0557*. Despite Test 2 providing the correct result (False = no SNP), Test1 suggests the presence of a mutation using a 25 nt probe (p = 0.0025). In this case, the 23 nt probe allowed us to confirm the absence of a SNP by a significant negative result (p = 0.9952). These results demonstrate how probe length can be used to optimize the detection of specific SNPs, if needed. Furthermore, we completed a global analysis by evaluating the effect of probe length for each hypothesis tested and we conclude that the use of two probe lengths for each hypothesis tested is redundant in the majority of cases (>99%).

#### Detection of mixed infections

**Fig 5:**
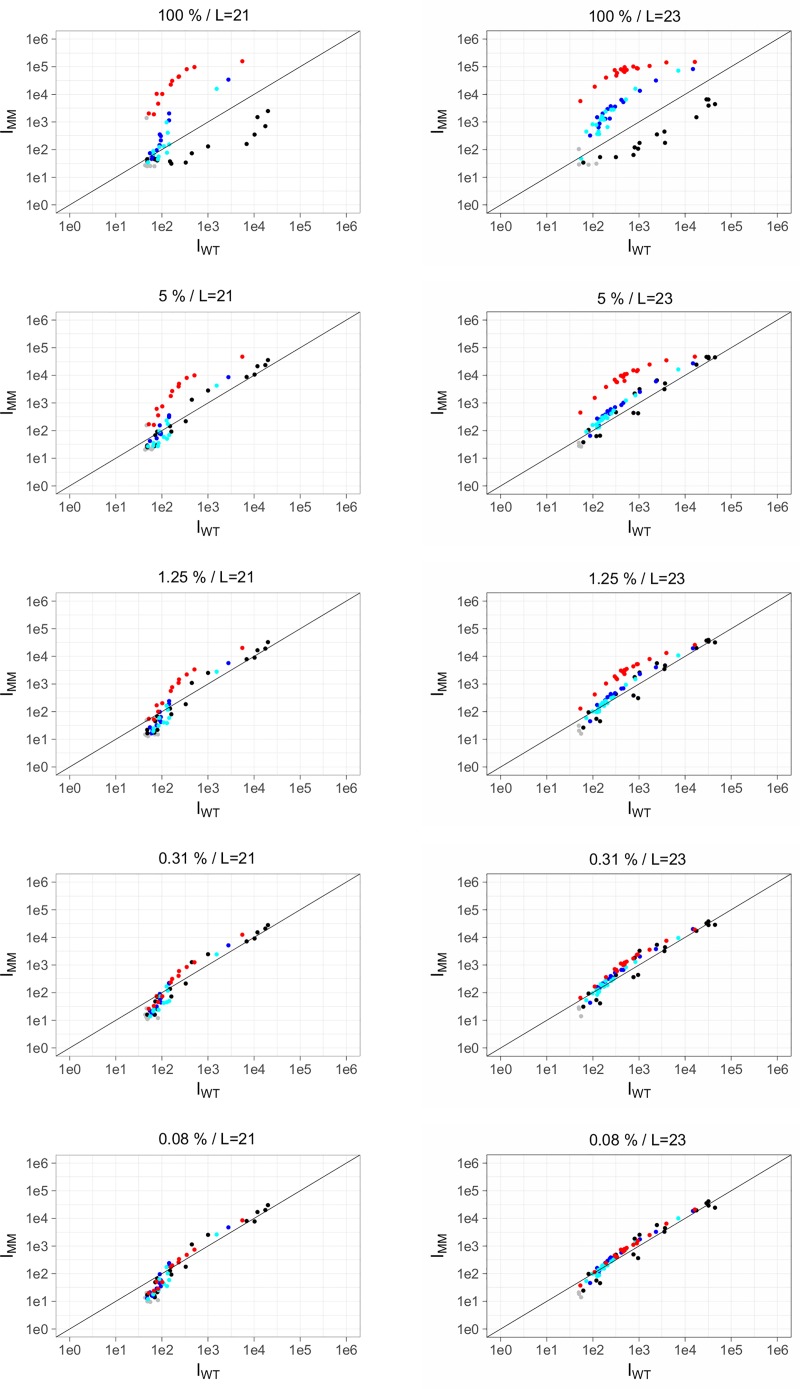
Detection of heteroresistance using probe sets targeting *rpoB* codon 531, SNP TCG > TTG (S531L). Probe lengths (L) of 21 or 23 nt long were used. A wild-type sample (CDC1511) and a mutant sample (strain 08–1074, Beijing genotype) were combined using the following mutant concentrations: 100%, 5%, 1.25%, 0.31% and 0.08%.

Given the clinical relevance of heteroresistance [23–27], we assessed the utility of our microarray to detect drug resistant *Mtb* in a mixed infection in which only a minority subpopulation of bacilli harbor resistance-conferring mutations. We performed an mPCR with samples containing a mixture of template DNA from WT (CDC1551) and 5%, 1.25%, 0.31% or 0.08% of an XDR-TB strain (strain 08–1074, Beijing genotype). This concentration range was chosen to determine the relative sensitivity of the microarray assay for the detection of resistant alleles in the context of a mixed infection. The use of an XDR-strain from a different strain-type background allowed us to evaluate detection of mutations with several probes sets in a single experiment. Based on analysis of probe sets targeting *rpoB* codon 531, we were able to detect drug resistance mutations even in the presence of ~80-fold excess WT DNA (1.25% MT concentration, **Fig 5**). The capability of our hybridization platform to detect the presence of low abundance mutations could be particularly valuable for early identification of drug resistance, before resistant clones come to dominate the infection. **Table 1** summarizes the results of the probe lengths comparison for the probe sets targeting mutations present in the XDR-TB strain tested in this experiment. The assay reliably detected the presence of mutations as low as 1.25% with some probe sets (**Table 1**). The inability to detect the *katG* 315 mutant allele when present at 5% of the population is consistent sequence context dependence of hybridization we have reported previously [22].

**Table 1:** Summary of drug resistance results for probe sets targeting differences between the WT and MT strain (08–1074).

% 08–1074	*rpoB codon 531* TCG > TTG		*katG codon 315* AGC > ACC		*rpsL codon 43* AAG > AGG		*embB codon 306* ATG > ATA	
	L = 21	L = 23	L = 23	L = 25	L = 23	L = 25	L = 23	L = 25
5	+	+	-	-	+	+	+	-
1.25	+	-	-	-	+	-	-	-
0.31	-	-	-	-	-	-	-	-
0.08	-	-	-	-	-	-	-	-

The plus (+) sign indicates the ability to identify the presence of the mutation with statistical significance. The minus (-) sign indicates the inability to detect the mutation by failure of any of the 3 statistical tests. L, probe length (nt).

## Discussion

In this study, we demonstrated the use of a 15-locus mPCR coupled with a custom, high-density microarray for robust detection of SNPs clinically relevant to TB diagnosis. This proof-of concept assay, capable of identifying 45 distinct alleles, was designed to enable simultaneous genotypic DST and strain typing of *Mtb*. Based on best estimates of clinical prevalence of drug resistance mutations, this prototype assay would detect >95% of rifampicin resistance, 70–90% of isoniazid resistance, and > 80% of strains resistance to second-line fluoroquinolones, as well as common mutations associated with resistance to additional clinically utilized therapeutics [28–31]. We tested this platform using *Mtb* clinical isolates from diverse lineages, including MDR and XDR strains, containing a variety of SNPs and successfully identified the presence/absence of SNPs in all cases.

Furthermore, we addressed the clinically relevant problem of detecting mixed *Mtb* infections, often referred to as heteroresistance. Heteroresistance can occur early during treatment as *Mtb* acquires drug resistance-conferring SNPs or upon reinfection with a drug-resistant strain [32]. Current methods are unable to detect low levels of drug-resistant bacilli which results in a delay of appropriate treatment. The WHO endorsed GeneXpert platform for detecting rifampin resistance, which relies on loss of signal in the presence of a MT, can only detect drug-resistant alleles when present in ~90% of the population [33]. According to a recent report, the new GeneXpert Ultra assay exhibited enhanced sensitivity for detection of heteroresistance for select *rpoB* mutations ranged from 10–40% mutant allele [34]. Performance for detection of other alleles from a mixed sample was not tested. In contrast, our hybridization approach enables the specific detection of MTs by utilizing probe sets specific to SNPs of interest enabling detection of MTs present at ~1.25% relative to WT (**Table 1**). This is a significant advantage for the early detection and treatment of drug-resistant *Mtb*. Similarly, this platform could also be designed to detect mixed infection involving multiple pathogens in addition to drug resistant variants of *Mtb*.

In the future, we can improve our assay by including probe sets for ~50 additional clinically relevant SNPs. In this proof-of-concept design, we included probe sets of two lengths for each targeted MT to enable robust identification of SNPs. We found in the majority of cases (>99%) the use of two probe sets was not required to identify the presence or absence of a SNP. Furthermore, our microarray included 5 technical replicates and 12–17 probes for each targeted MT enabling a high dynamic range and ability to differentiate SNPs. These features allow for the specific identification of the MT present and the ability to identify MTs present in a minor population of target analyte. The importance of multiple probes for detection of a SNP is highlighted by the ability of our platform to specifically identify mutations despite the presence of polymorphisms present in the same or nearby codons (**Table C in S1 File, A**, in blue). This can occur due to strain dependent genetic variants unrelated to drug resistance or unknown SNPs conferring resistance not targeted by probes that could interfere with hybridization based detection [35, 36]. In this scenario, there is a global loss of intensity with all probe sets but the relative intensity difference between the branches remains the same enabling the identification of a SNP. This is a major advantage of our platform compared to current hybridization based platforms for the detection of drug resistance. However, if this level of sensitivity is not required, the number of probe sequence variations for each set can be decreased to allow for the detection of even more clinically relevant SNPs.

## Conclusions

We have demonstrated the use of this methodology as a promising alternative to current molecular methods for DST and strain typing for *Mtb*. We envision positioning of a diagnostic tool for high-resolution SNP analysis of DST and strain typing based on this proof-of-concept platform as a second-line assay following initial detection of *Mtb*. Miniaturization of this hybridization based platform as a lab-on-chip product could be amenable for front-line use. The principles of the current fluorescence-based approach can in principle be transferred to other methods such as impedimetric biosensors or surface plasmon resonance interfaces [37, 38]. The data analysis is robust and can be automated, making the methodology simpler compared to next-generation sequencing approaches. Other groups have utilized microarray-based approaches, but are limited in their coverage of clinically relevant SNPs and use fewer probes per SNP [39, 40]. A large number of probes are not always necessary for each SNP, as this depends on the dynamic range in each case. However, this method ensures that also minor mutations (from a thermodynamic point of view) can be detected accurately. The methodology presented here for the detection of SNPs offers a novel alternative for the detection of *Mtb*, strain type, and drug susceptibility with the potential to provide next day results. Recently, our hybridization-based approach for the detection of SNPs was applied to the simultaneous detection of a limited number of clinically relevant KRAS oncogene point mutations [22]. This study further validates and extends our platform for the robust detection of clinically relevant SNPs as a valuable diagnostic tool for a variety of priority health concerns ranging from cancer to infectious disease.

## Supporting information

S1 File **Table A. Multiplex PCR primers. Table B. Example of probe set design. Table C. Summary of drug resistance and strain typing results for all strains tested. Figure A. Agarose gel validation of fifteen loci multiplex PCR.** Lane M is a 2 log DNA ladder (NEB) and lane 1 is the mPCR profile of WT Mtb DNA (CDC1551 DNA). The white arrows indicate nonspecific PCR products. This gel is representative of all mPCR products used in this study.**Figure B. Illustration of statistical tests. A)** Example where the minimal distance (red arrow) of the mutant branch (red dots) from the LOWESS curve (black curve) is higher than the p75 quantile distances of the reference branch (black dots) with the LOWESS curve. The black arrows represent the distances of each spot of the reference branch from the LOWESS curve, from which the p75 quantile is subsequently calculated. **B)** Same as in A, but here the minimal distance of the mutant branch from the LOWESS curve is too small and Test 2 is rejected. **C)** Example where the median distance (red arrow) of the mutant branch (red dots) from the LOWESS curve (black curve) is higher than the median distance (blue or cyan arrow) of each side branch (blue and cyan dots) from the LOWESS curve. **D)** Same as C, but here the median distance of the mutant branch is smaller than the median distances of the side branches, thus Test 3 is rejected.
(DOCX)
