# Supplementary material for: Molecular drug susceptibility testing and strain typing of tuberculosis by DNA hybridization
Source: PLoS One. 2019 Feb 7;14(2):e0212064. doi: 10.1371/journal.pone.0212064 (PMC6366778; doi:10.1371/journal.pone.0212064)
Supplement: S1 File — Table A. Multiplex PCR primers. Table B. Example of probe set design. Table C. Summary of drug resistance and strain typing results for all strains tested. Figure A. Agarose gel validation of fifteen loci multiplex PCR. Lane M is a 2 log DNA ladder (NEB) and lane 1 is the mPCR profile of WT Mtb DNA (CDC1551 DNA). The white arrows indicate nonspecific PCR products. This gel is representative of all mPCR products used in this study.Figure B. Illustration of statistical tests. A) Example where the minimal distance (red arrow) of the mutant branch (red dots) from the LOWESS curve (black curve) is higher than the p75 quantile distances of the reference branch (black dots) with the LOWESS curve. The black arrows represent the distances of each spot of the reference branch from the LOWESS curve, from which the p75 quantile is subsequently calculated. B) Same as in A, but here the minimal distance of the mutant branch from the LOWESS curve is too small and Test 2 is rejected. C) Example where the median distance (red arrow) of the mutant branch (red dots) from the LOWESS curve (black curve) is higher than the median distance (blue or cyan arrow) of each side branch (blue and cyan dots) from the LOWESS curve. D) Same as C, but here the median distance of the mutant branch is smaller than the median distances of the side branches, thus Test 3 is rejected. (DOCX) [file pone.0212064.s001.docx]

**Molecular Drug Susceptibility Testing and Strain Typing of Tuberculosis**

**by DNA Hybridization**

**Hillary N. Wood^1^**¶**, Tom Venken^2^** ¶,#a,#b**, Hanny Willems^2^, An Jacobs^2^, Ana Júlia Reis^3^, Pedro Eduardo Almeida da Silva^3^, Susanne Homolka^4,5^, Stefan Niemann^4,5^, Kyle H. Rohde^1,*^, and Jef Hooyberghs^2,6^**

^1^ Division of Immunity and Pathogenesis, Burnett School of Biomedical Sciences, College of Medicine, University of Central Florida, Orlando, Florida

^2^ Flemish Institute for Technological Research, VITO, Mol, Belgium

^3^ Laboratory of Tuberculosis, Faculty of Medicine, Universidade Federal do Rio Grande- FURG, Brazil

^4^ Molecular and Experimental Mycobacteriology, Research Center Borstel, Borstel, Germany

^5^ German Center for Infection Research, Borstel, Germany

^6^ Theoretical Physics, Hasselt University, Diepenbeek, Belgium

#^a^ Current Address: ﻿VIB Center for Cancer Biology, Leuven, Belgium

#^b^ Current Address: ﻿Laboratory for Translational Genetics, Department of Human Genetics, KU Leuven, Leuven, Belgium

* Corresponding author

E-mail: [kyle.rohde@ucf.edu](mailto:kyle.rohde@ucf.edu)

¶ These authors contributed equally to this work.

Table of Contents

[Table A - Multiplex PCR Primers 3](#_Toc518645048)

[Figure A. Agarose gel validation of fifteen loci multiplex PCR. 5](#_Toc518645049)

[Probe Set Design 6](#_Toc518645050)

[Table B: Example of probe set design. 8](#_Toc518645051)

[Statistical Tests 9](#_Toc518645052)

[Figure B. Illustration of statistical tests. 10](#_Toc518645053)

[Table C- Summary of drug resistance and strain typing results for all strains tested. 11](#_Toc518645054)

[Drug Resistance Schemes & Scatterplots 12](#_Toc518645055)

[08-1074 12](#_Toc518645056)

[07-2328 13](#_Toc518645057)

[09-3657 14](#_Toc518645058)

[08-0757 15](#_Toc518645059)

[07-3082 16](#_Toc518645060)

[07-3216 17](#_Toc518645061)

[08-1186 18](#_Toc518645062)

[03-9532 19](#_Toc518645063)

[03-4850 20](#_Toc518645064)

[Strain Typing Schemes & Scatterplots 21](#_Toc518645065)

[08-1074 21](#_Toc518645066)

[07-2328 22](#_Toc518645067)

[09-3657 23](#_Toc518645068)

[07-3082 24](#_Toc518645069)

[07-3216 25](#_Toc518645070)

[08-1186 26](#_Toc518645071)

[03-9532 27](#_Toc518645072)

[03-4850 28](#_Toc518645073)

# Table A - Multiplex PCR Primers

| **Locus** | **Primer^a^** | **Sequence (5' to 3')^b^** | **Size (bp)** | **Tm (°C)** | **Product size (bp)** | **[Primer] (nM)** |
| --- | --- | --- | --- | --- | --- | --- |
| **Identification loci** | | | | | | |
| *16S rRNA* | 16s_rRNA_F | ggcgtgcttaacacatgca | 17 | 66 | 276 | 147 |
|  | 16s_rRNA_R | *CTGGCGTCATAGCTGTTTCCTGTGTGA*ccggctacccgtcgtc | 18 | 66 |  |  |
| *23S rRNA* | 23s_rRNA_F | ctctgctgccaagaaaagcc | 20 | 66 | 437 | 147 |
|  | 23S_rRNA_R | *CTGGCGTCATAGCTGTTTCCTGTGTGA*caggtcggaacttacccgac | 20 | 66 |  |  |
| **Drug resistance loci** | | | | | | |
| *embB* | embB_F | gctgaaactgctggcgatc | 19 | 66 | 527 | 147 |
|  | embB_R | *CTGGCGTCATAGCTGTTTCCTGTGTGA*cgggtttgctggcctc | 16 | 66 |  |  |
| *gyrA* | gyrA_F | gactcgctcgaccggatc | 18 | 66 | 397 | 147 |
|  | gyrA_R | *CTGGCGTCATAGCTGTTTCCTGTGTGA*gggcttcggtgtacctcatc | 20 | 66 |  |  |
| *inhA* | inhA(P)_F | gcggatgatgtcagcgg | 17 | 67 | 1018 | 1176 |
|  | inhA(P)_R | CTGGCGTCATAGCTGTTTCCTGTGTGAacgaatgggggtttggc | 19 | 65 |  |  |
| *katG* | katG_F | atggccatgaacgacgtc | 18 | 65 | 326 | 147 |
|  | katG_R | *CTGGCGTCATAGCTGTTTCCTGTGTGA*gtgtattgccaagcgcca | 18 | 66 |  |  |
| *pncA* | pncA_F | cagctggtcatgttcgcg | 18 | 67 | 615 | 294 |
|  | pncA_R | CTGGCGTCATAGCTGTTTCCTGTGTGA gctgtcaggtccaccagc | 18 | 65 |  |  |
| *rpoB* | rpoB_F | gtcgccgcgatcaaggagtt | 20 | 71 | 230 | 147 |
|  | rpoB_R | *CTGGCGTCATAGCTGTTTCCTGTGTG*Accctcaggggtttcgatcggg | 21 | 74 |  |  |
| *rpsL* | rpsL_F | gtagcgccctgcttcgg | 17 | 67 | 689 | 294 |
|  | rpsL_R | *CTGGCGTCATAGCTGTTTCCTGTGTGA*caacggacgcttgggc | 16 | 67 |  |  |
| *rrs* | rrs_F | cgatgcaacgcgaagaac | 18 | 66 | 744 | 294 |
|  | rrs_R | *CTGGCGTCATAGCTGTTTCCTGTGTGA*ccttagaaaggaggtgatccagc | 23 | 66 |  |  |
|  | | | | | | |
|  | | | | | | |
| **Strain typing loci** | | | | | | |
| ***Rv0129c*** | Rv0129c_F | cacgcggtctacctgctc | 18 | 65 | 352 | 147 |
|  | Rv0129c_R | *CTGGCGTCATAGCTGTTTCCTGTGTGA*gctgcgggtagtacgcg | 17 | 66 |  |  |
| ***Rv0557*** | Rv0557_F | gacggtgtccgggtgc | 16 | 67 | 961 | 1176 |
|  | Rv0557_R | *CTGGCGTCATAGCTGTTTCCTGTGTGA*cctcgtagtggccgagc | 17 | 65 |  |  |
| ***Rv1009*** | Rv1009_F | ggacccaggatgtgacgttc | 20 | 67 | 469 | 147 |
|  | Rv1009_R | *CTGGCGTCATAGCTGTTTCCTGTGTGA*ctctttggccagccgc | 16 | 66 |  |  |
| ***Rv1811*** | Rv1811_F | cttgcgatcgggaaggactc | 20 | 68 | 565 | 294 |
|  | Rv1811_R | *CTGGCGTCATAGCTGTTTCCTGTGTGA*gtgcgctgcacgatatgg | 18 | 67 |  |  |
| ***Rv2629*** | Rv2629_F | gagctcatcgacagcctcg | 19 | 66 | 835 | 294 |
|  | Rv2629_R | *CTGGCGTCATAGCTGTTTCCTGTGTGA*catcggcccttgccac | 16 | 67 |  |  |

^a^F: forward primer, R: reverse primer

^b^Barcode sequences of the reverse primers are shown in uppercase

**
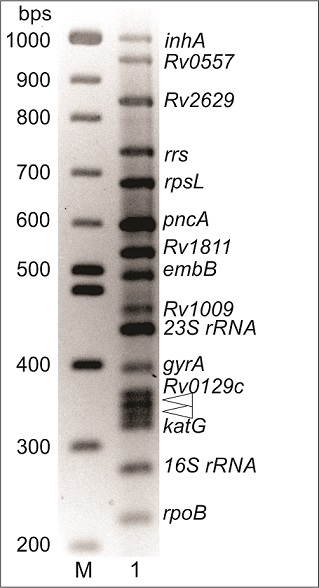
**

Figure A. Agarose gel validation of fifteen loci multiplex PCR.

# Probe Set Design

In this study, for each targeted MT we designed a probe set to test a specific hypothesis: the presence or absence of a SNP at a defined nt position. Probe sets were designed based on previous data from the Hooyberghs laboratory (21). Probes for SNP discrimination are designed by combining nucleotide (nt) sequences and probe length variations that correspond to the MT of interest (16). Hadiwikarta *et al.* demonstrated that an optimal dynamic range is reached when probe lengths of 21 to 25 nt are used (16). These lengths allow specificity for SNPs and are sufficiently long to avoid end effects; i.e. mismatches close to the end of a double helix are less destabilizing compared to mismatches near the middle. In addition, the use of multiple probe lengths provides enough redundancy in the assay to confidently identify SNPs and the ability to compare the dynamic range of the fluorescence intensities resulting from each probe set. The selection of probe length for each hypothesis tested is based on a theoretical estimation of the free energy for DNA duplex formation. In **Fig 5** we have investigated how probe length affects the ability to identify the presence/absence of a MT.

For this proof-of-concept study we used an Agilent 8x15k microarray slide. As a result, the number of possible probes is restrained to a maximum of 15,208. Each hypothesis was tested using 5 technical replicates, therefore the number of unique probes on the chip is reduced from 15,208 to 3,030. The use of 2 probe sets with different lengths for each SNP (either 21 and 23 nt long or 23 and 25 nt long, depending on the predicted hybridization free energy in each locus of interest) further reduces the available probes per set to 1,515 probes. Initially we designed probe sets for wild-type (WT) and the 3 alternative nt substitutions (SNPs) at each defined location associated with drug resistance. MTs that are not associated with drug resistance, i.e. silent MTs, were omitted from the drug resistance scheme (**Fig 3C**). In the case of strain typing, probe sets were designed to target only wild-type or the single alternative nt substitution which allows for strain typing, as the latter is well defined (10). We aimed to use 17 probes for the reference branch and 17 for the mutant branch in each set (branch definitions are explained more in Materials and Methods, Data Analysis) to obtain a broad dynamic range, using sequences as illustrated in Table S2. This results in approximately 35 probes (17 probes x 2 branches) for each strain typing set, or a total of 350 probes for each strain typing experiment on the whole array by including 5 technical replicates and 2 different probe lengths. In addition to strain typing loci, specific detection of *Mtb* is also feasible by including 16S rRNA and 23S rRNA mPCR amplicons which contain hypervariable regions enabling the specific detection of *Mycobacterium tuberculosis*. However, probe sets for 16S rRNA and 23S rRNA were not included on the microarray since the platform was focused on the differentiation of single nucleotide polymorphisms.

In comparison to strain typing experiments, drug resistance experiments require twice as many probes due to the inclusion of side branches (also alternative SNPs on the same location were tested). Therefore 68 probes per set are needed (14-17 probes x 4 branches) or a total 680 for each drug resistance hypothesis on the whole array including replicates and 2 different probe lengths.

As an example, to illustrate the design process, the probe set for the detection of the SNP (C>T) in nt 221 of *Rv0557* which is specific for *M. africanum* 1a/1b is shown in **Table B**. Probes are 25 nt long with the MT of interest placed in the middle of the sequence (13^th^ nt). The WT probe is indicated as perfect match (PM) and is used as a template to design additional probes. All other probes in the set contain either 1 mismatch (1MM) or 2 mismatches (2MM) in defined locations based on a rational design which accounts for the relative free energy penalty of the MT. The combination of the PM, 1MM and 2MM probes allows for sufficient dynamic range to identify the presence of SNPs. Note, all MTs introduced in probes are at least 4 bps from the ends to avoid non-additive free energy penalties as shown by Hadiwikarta *et al.*(17).

Table B: Example of probe set design.

| Type | Sequence |
| --- | --- |
| Target sequence |  |
| Wild-type (target) | 5’-CAAAGGTGACCA C GTTGCCGCTCGG-3’ |
|  |  |
| Probe sequences |  |
| PM | 3’-GTTTCCACTGGT G CAACGGCGAGCC-5’ |
|  |  |
| 1 MM | 3’-GTTTCCACTGGT **A** CAACGGCGAGCC-5’ |
|  | 3’-GTTT**A**CACTGGT G CAACGGCGAGCC-5’ |
|  | 3’-GTTT**T**CACTGGT G CAACGGCGAGCC-5’ |
|  | 3’-GTTT**G**CACTGGT G CAACGGCGAGCC-5’ |
|  | 3’-GTTTC**A**ACTGGT G CAACGGCGAGCC-5’ |
|  | 3’-GTTTC**T**ACTGGT G CAACGGCGAGCC-5’ |
|  | 3’-GTTTC**G**ACTGGT G CAACGGCGAGCC-5’ |
|  |  |
| 2 MM | 3’-GTTT**A**CACTGGT **A** CAACGGCGAGCC-5’ |
|  | 3’-GTTT**A**CACT**C**GT G CAACGGCGAGCC-5’ |
|  | 3’-GTTT**A**CACT**T**GT G CAACGGCGAGCC-5’ |
|  | 3’-GTTT**A**CACT**A**GT G CAACGGCGAGCC-5’ |
|  | … |

The wild-type (WT) target sequence is used as a template to design the perfect match (PM), single mismatch (1MM) and double mismatch (2MM) probe sequences. MTs are indicated as underlined text.

# Statistical Tests

To confirm the presence of specific SNPs with high statistical confidence and prevent detection of false-positives, we used locally weighted scatterplot smoothing (LOWESS), which determines the vertical distance between the reference branch and all side branches using Wilcoxon ranking. Test 1 determines the p-value of the vertical distance between the mutant branch and the reference or side branches is below 0.05. If the p-value is high (> 0.05) the presence of a MT cannot be confirmed. Test 2 determines if the minimal vertical distance of the mutant branch is greater than the 75^th^ percentile (p75 quantile) of the distances of the reference branch from the LOWESS curve, as illustrated in **Fig S2A and S2B**. This test will not include a MT when the branch points lie too close to each other. Note that the LOWESS curve is omitted in most of the figures in this manuscript for simplicity. Finally, Test 3 compares the median of the mutant branch and alternate side branch vertical distances from the LOWESS curve. If the median distance of the mutant branch is greater than this of alternate side branches the MT can be confirmed (see **Fig S2C**), otherwise the hypothesis is proven false (**Fig S2D**). Note that for strain typing experiments only Test 1 and Test 2 are applied. Test 3 is invalid because only one MT at each position is evaluated resulting in a single deviating branch (mutant branch).


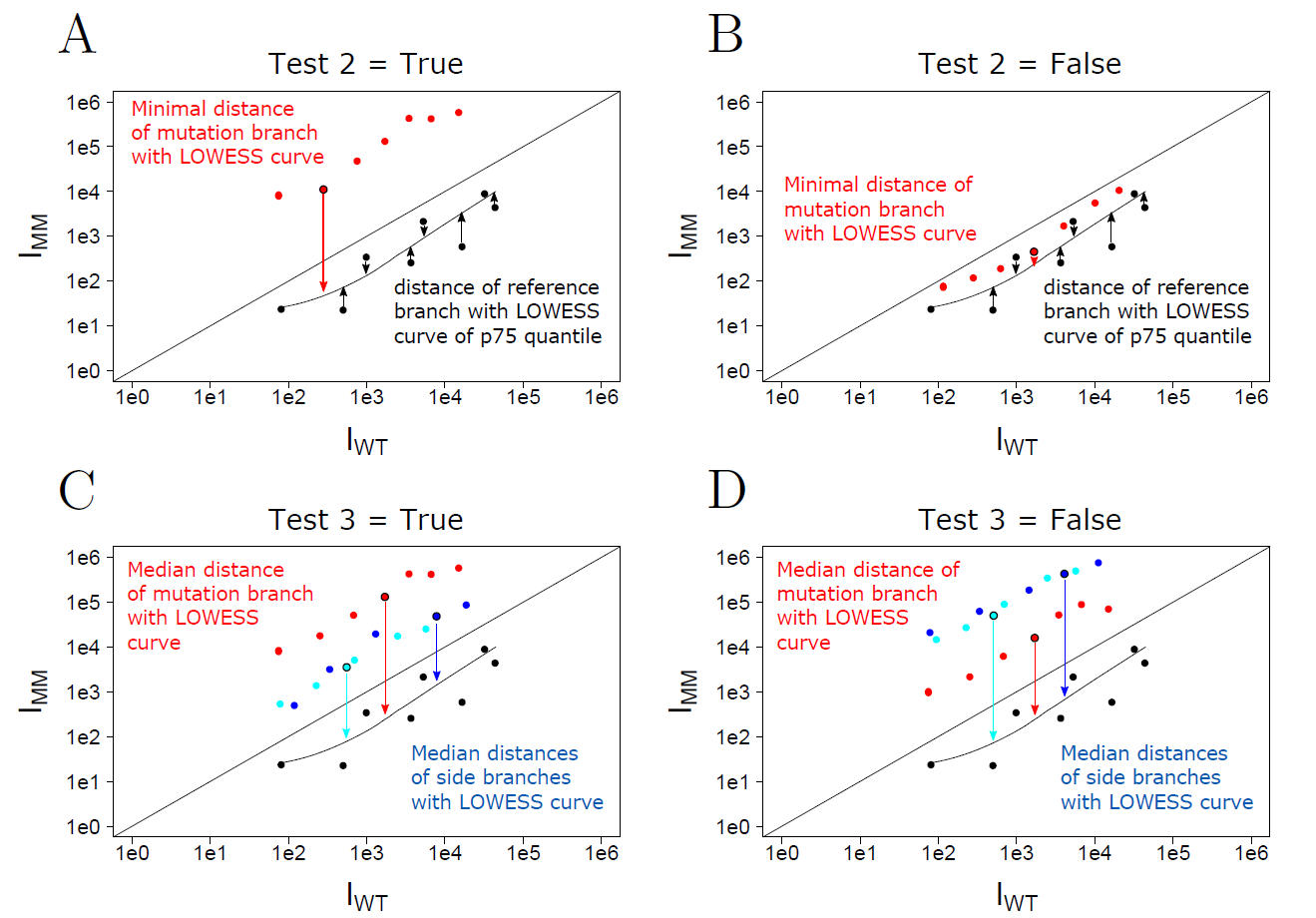


Figure B. Illustration of statistical tests.

Table C - Summary of drug resistance and strain typing results for all strains tested.

The table indicates the presence or absence of SNPs in each target loci as confirmed by sequencing for ^a^drug resistance or ^b^strain typing. If no SNP was detected by the microarray and the sequence is wild-type (WT) this is indicated in the table as WT. If a MT was found by the microarray and confirmed by sequencing this is notated in black text with the codon number followed by the specific nucleotide substitution. In some cases, drug resistance MTs were found by sequencing, but not targeted by probe sets on the microarray and are indicated by blue text.

# Drug Resistance Schemes & Scatterplots

### 08-1074

**
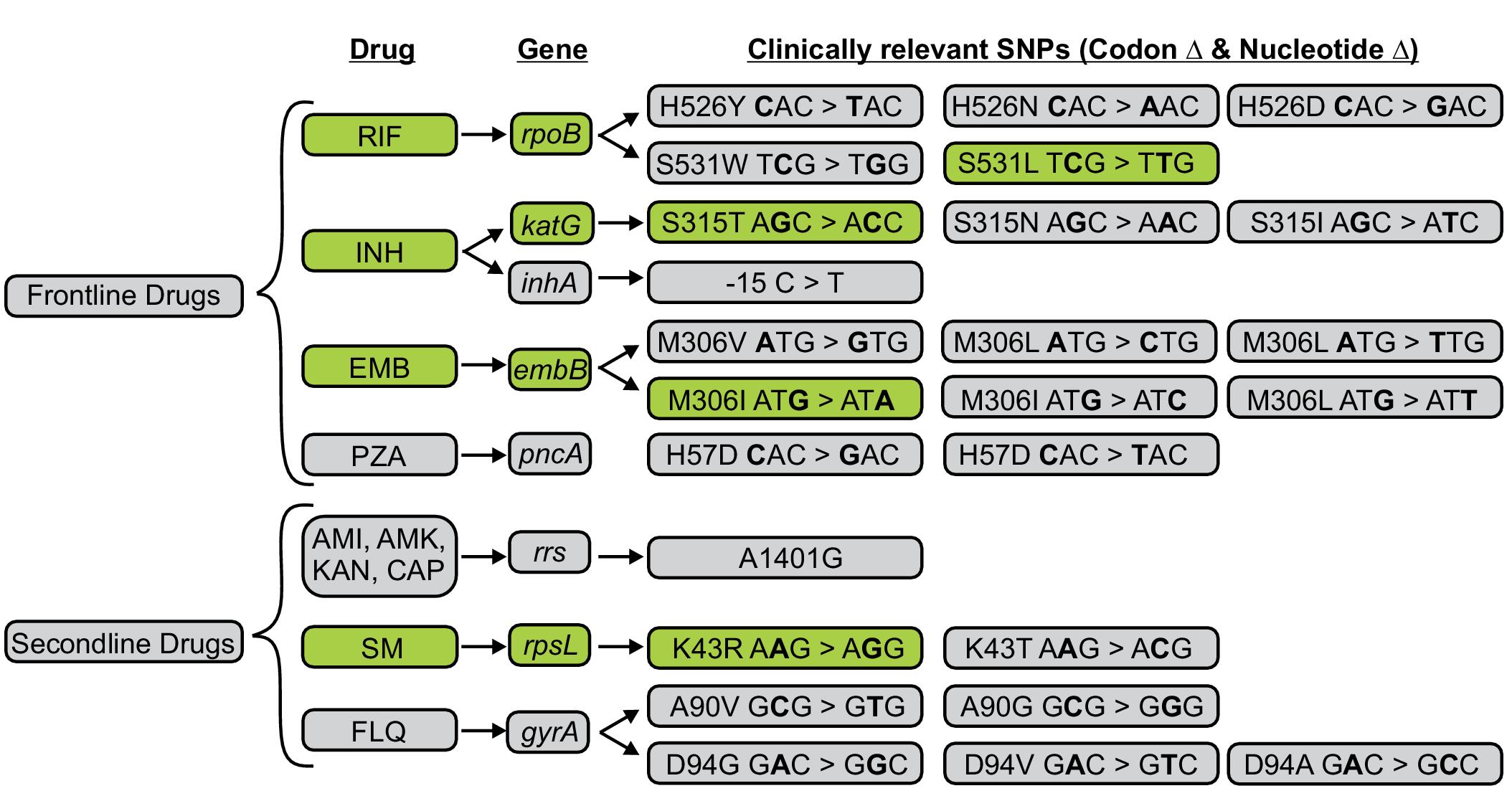
**

**
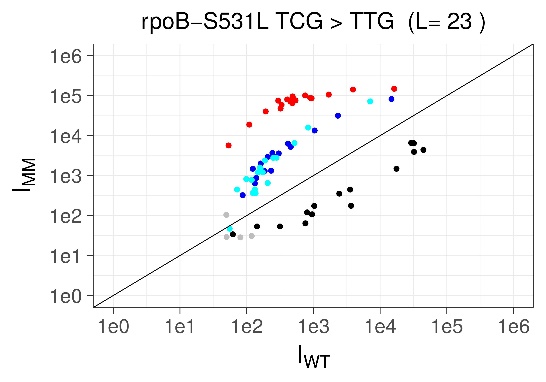

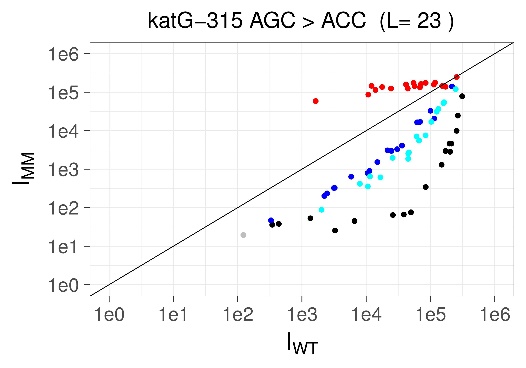

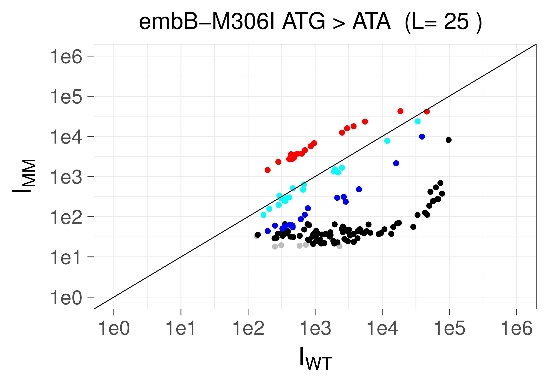

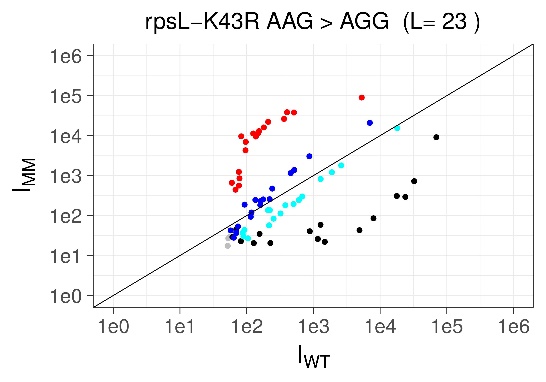
**

### 07-2328

**
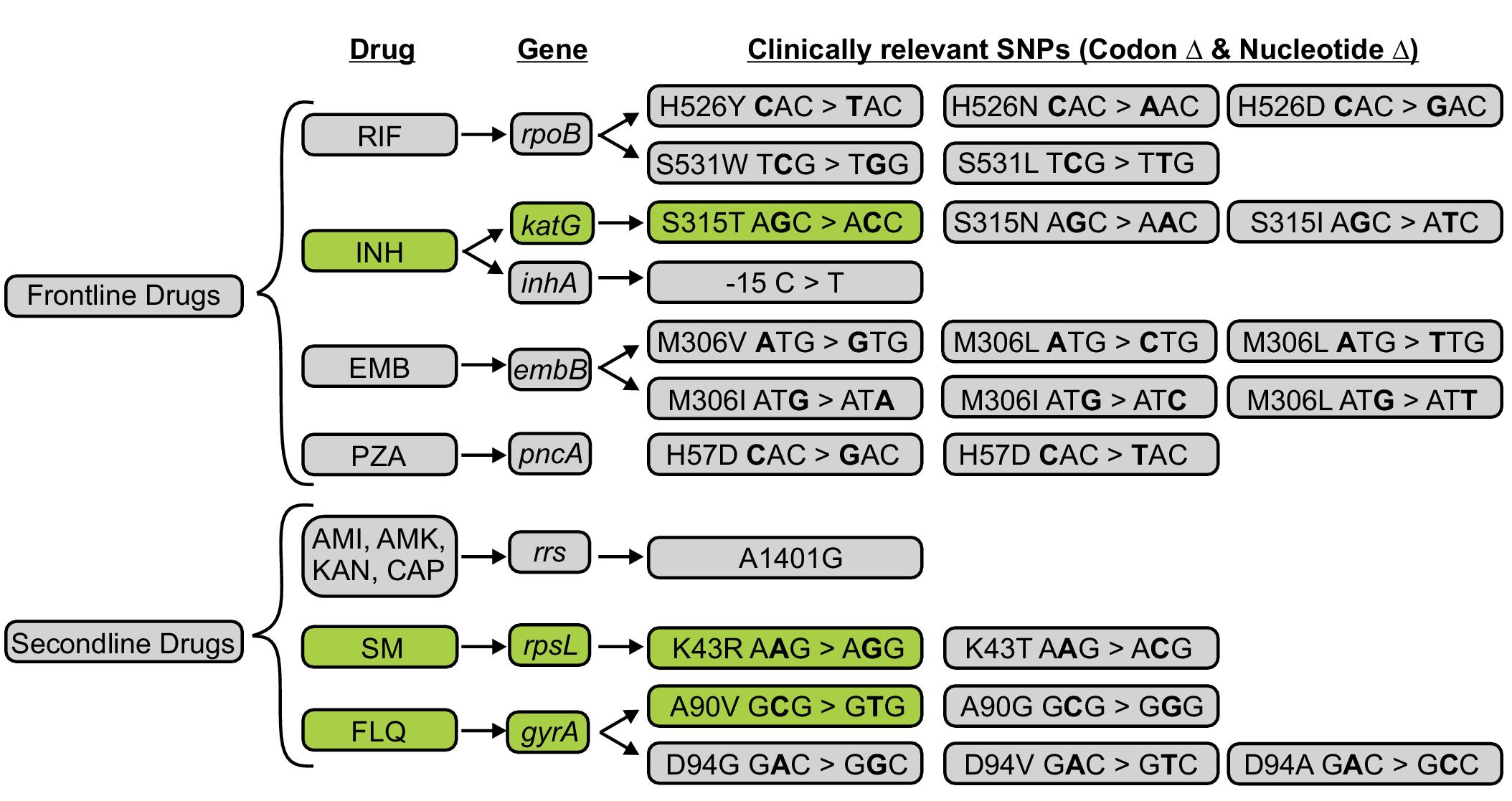
**

**
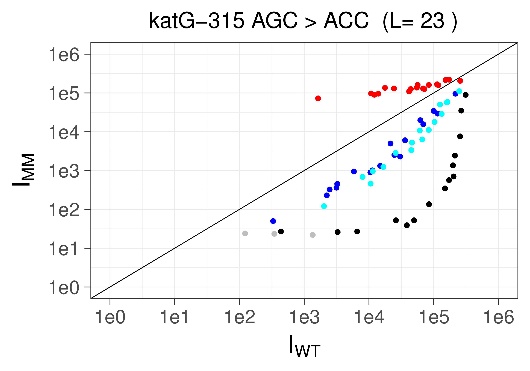

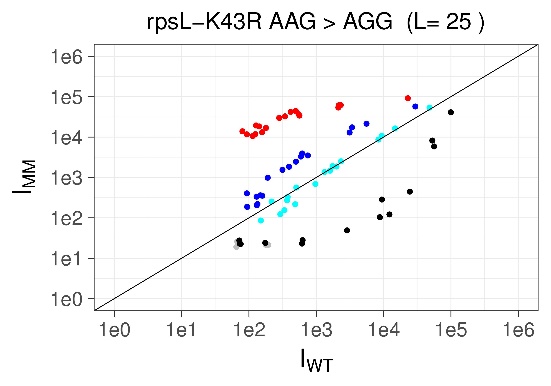

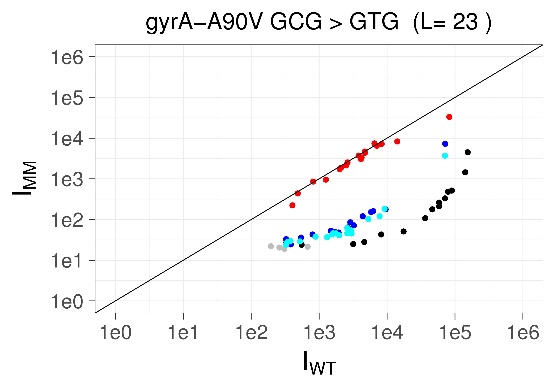
**

### 09-3657


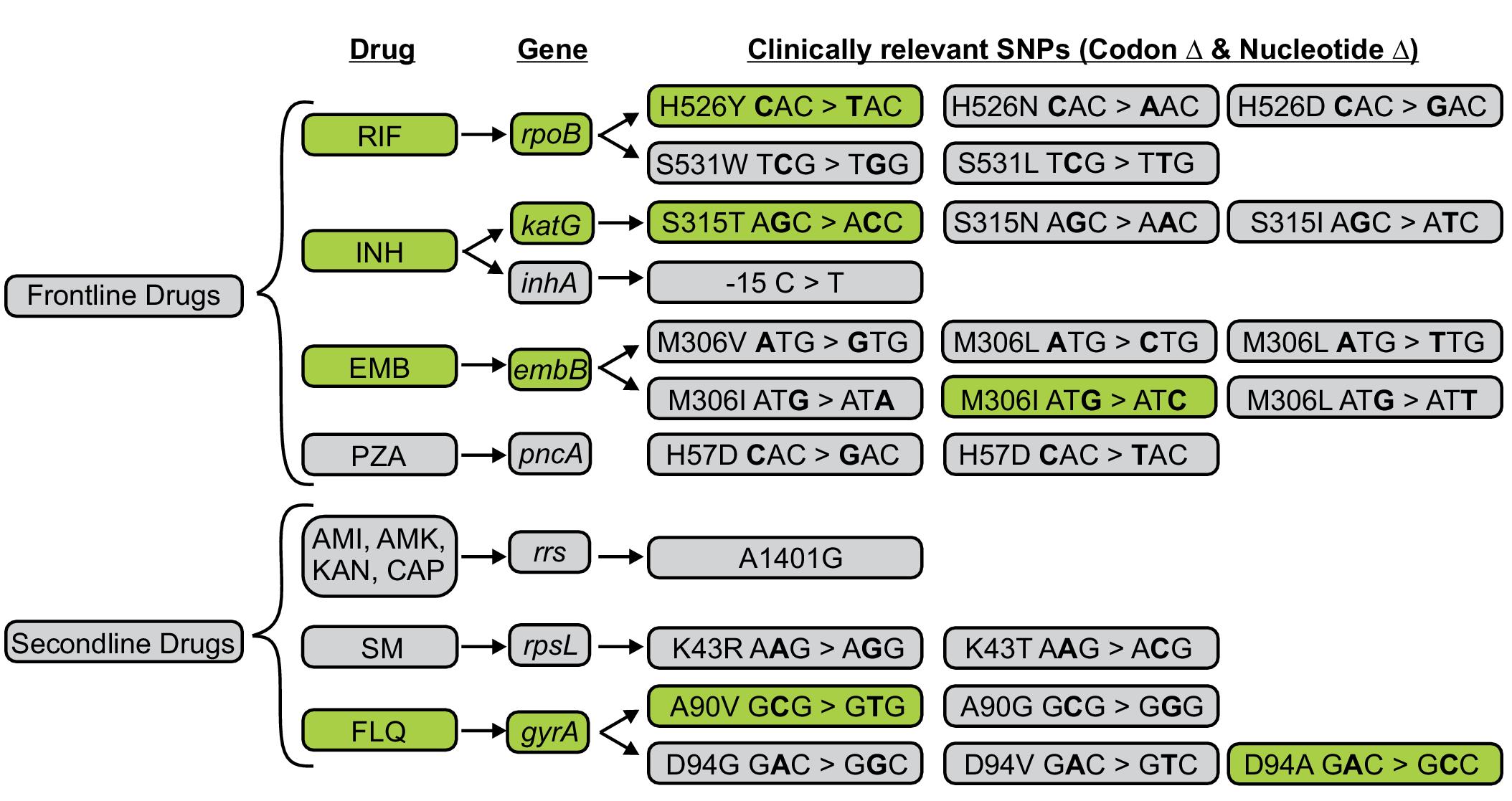


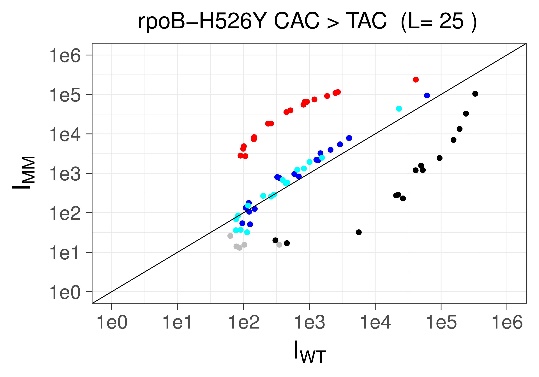

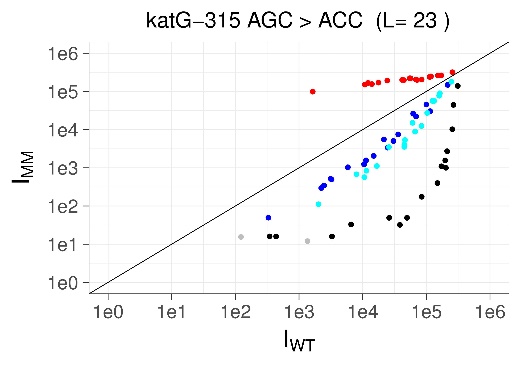

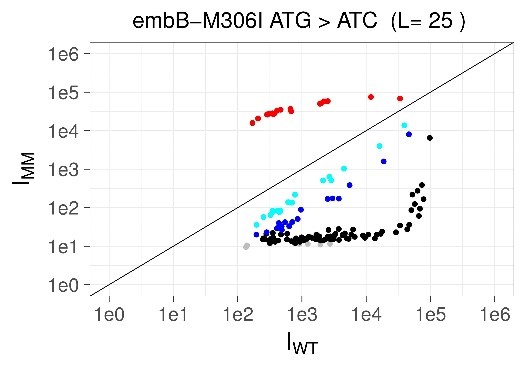

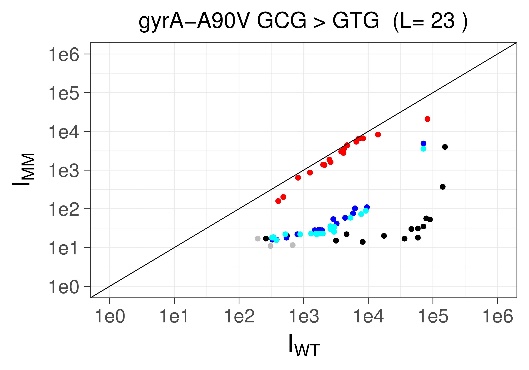

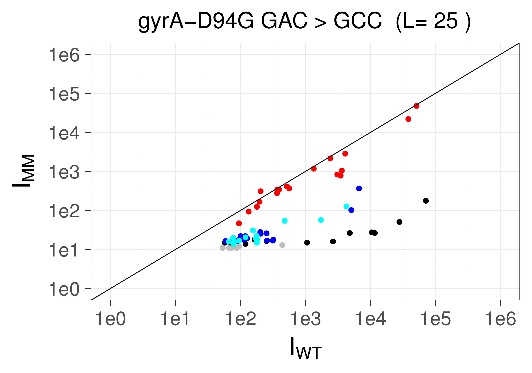


### 08-0757


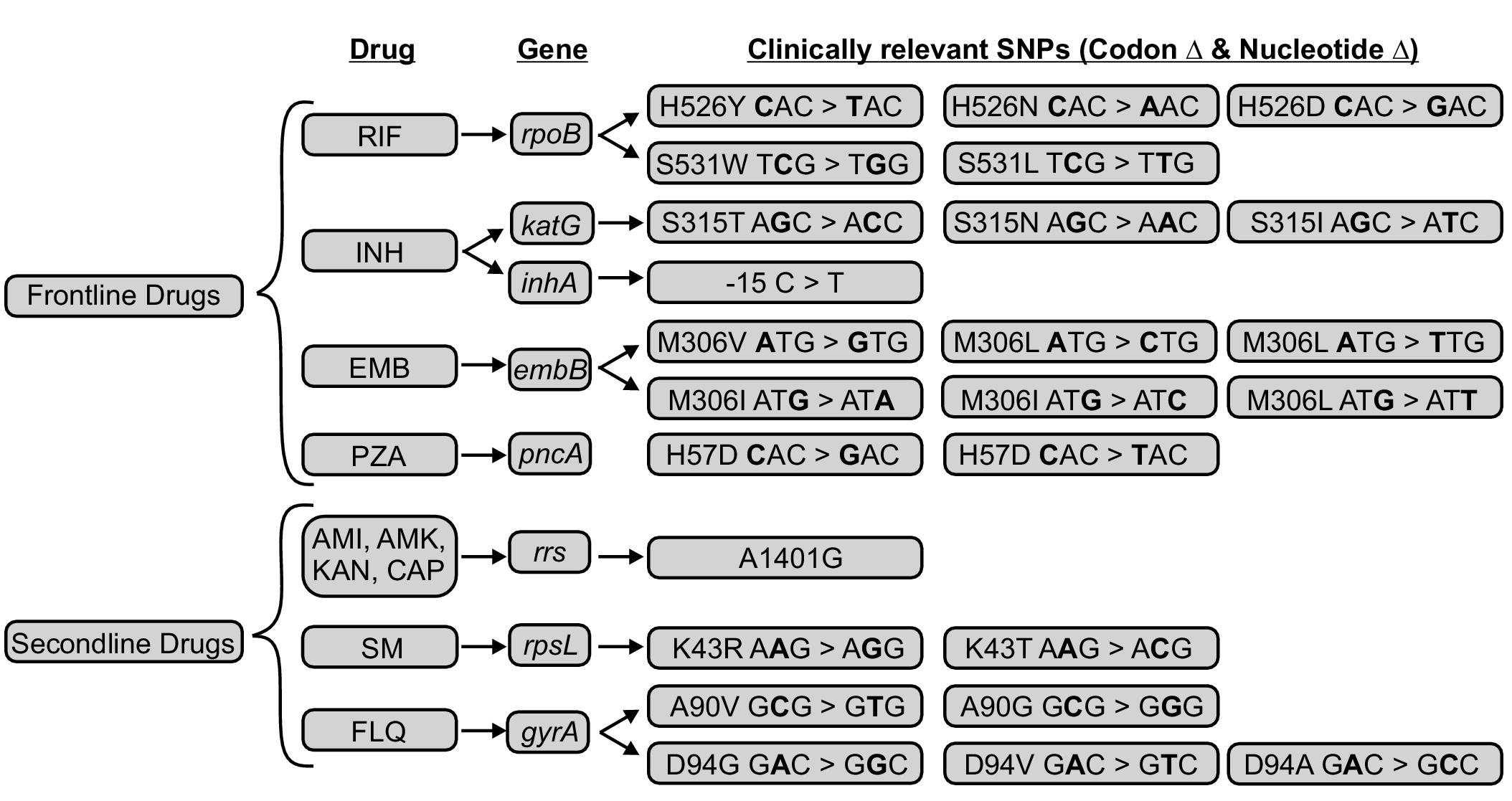


### 07-3082

**
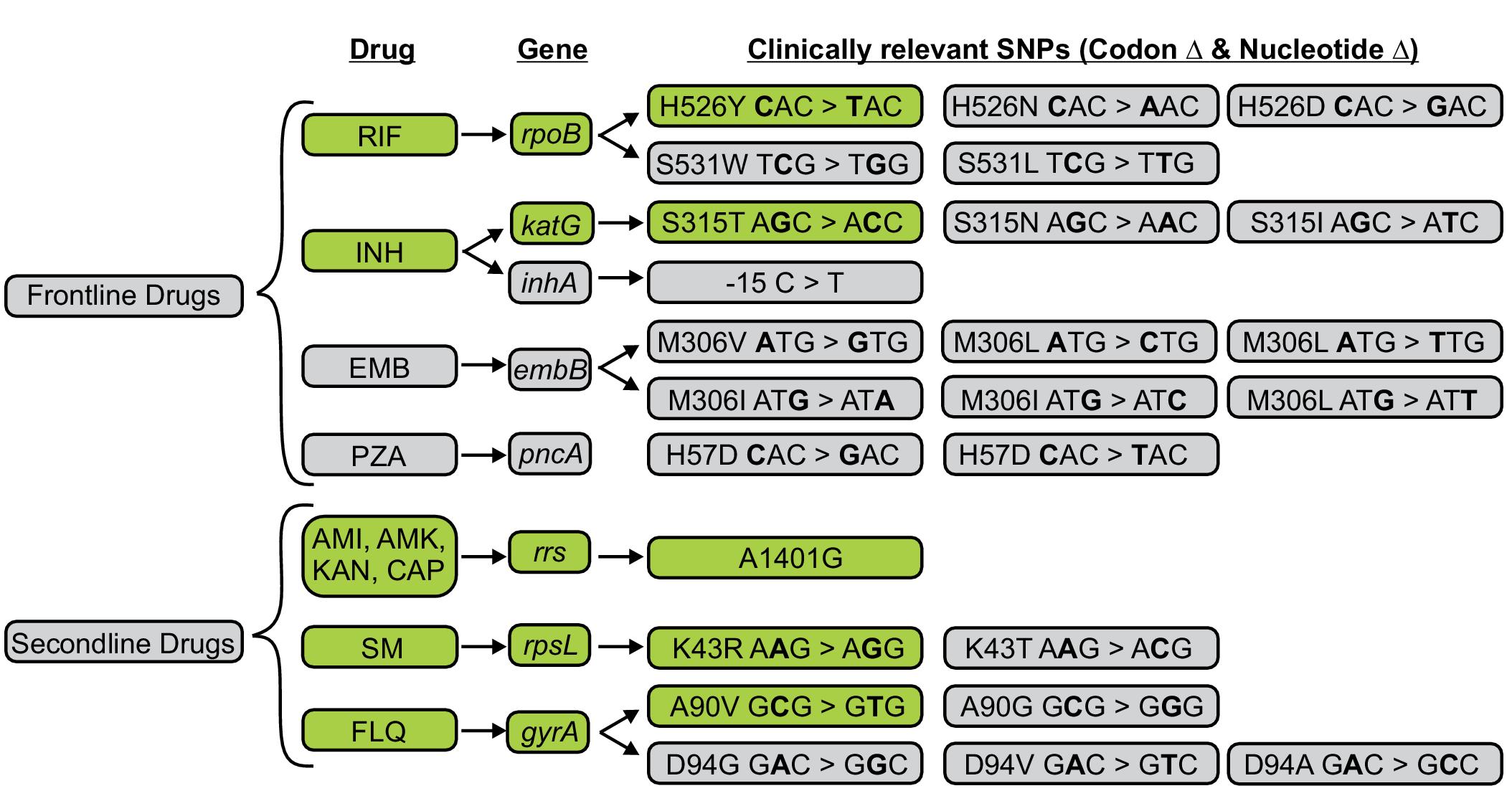
**


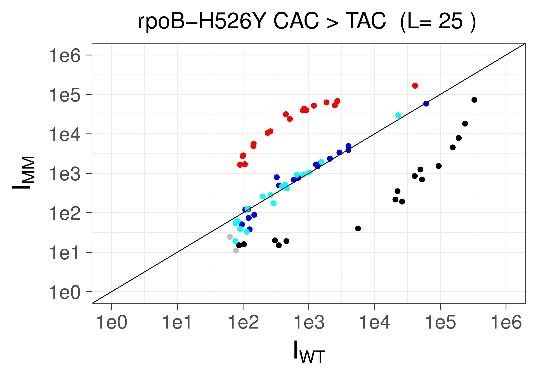

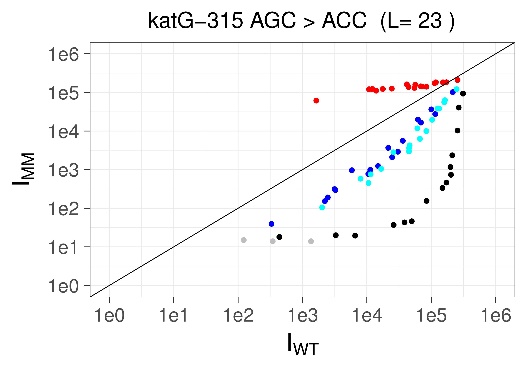

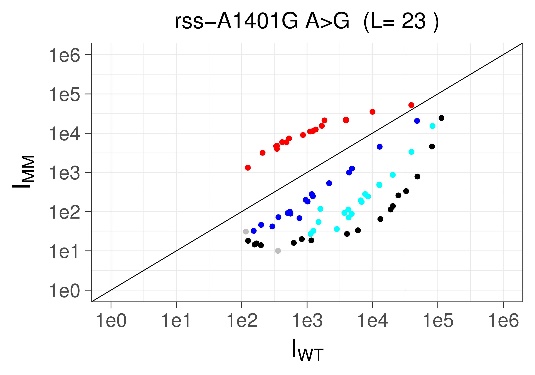


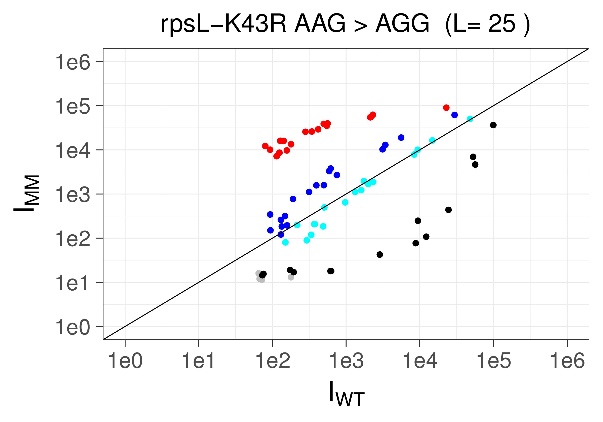

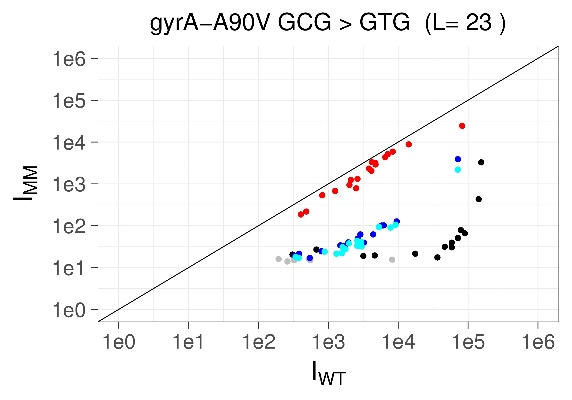


### 07-3216


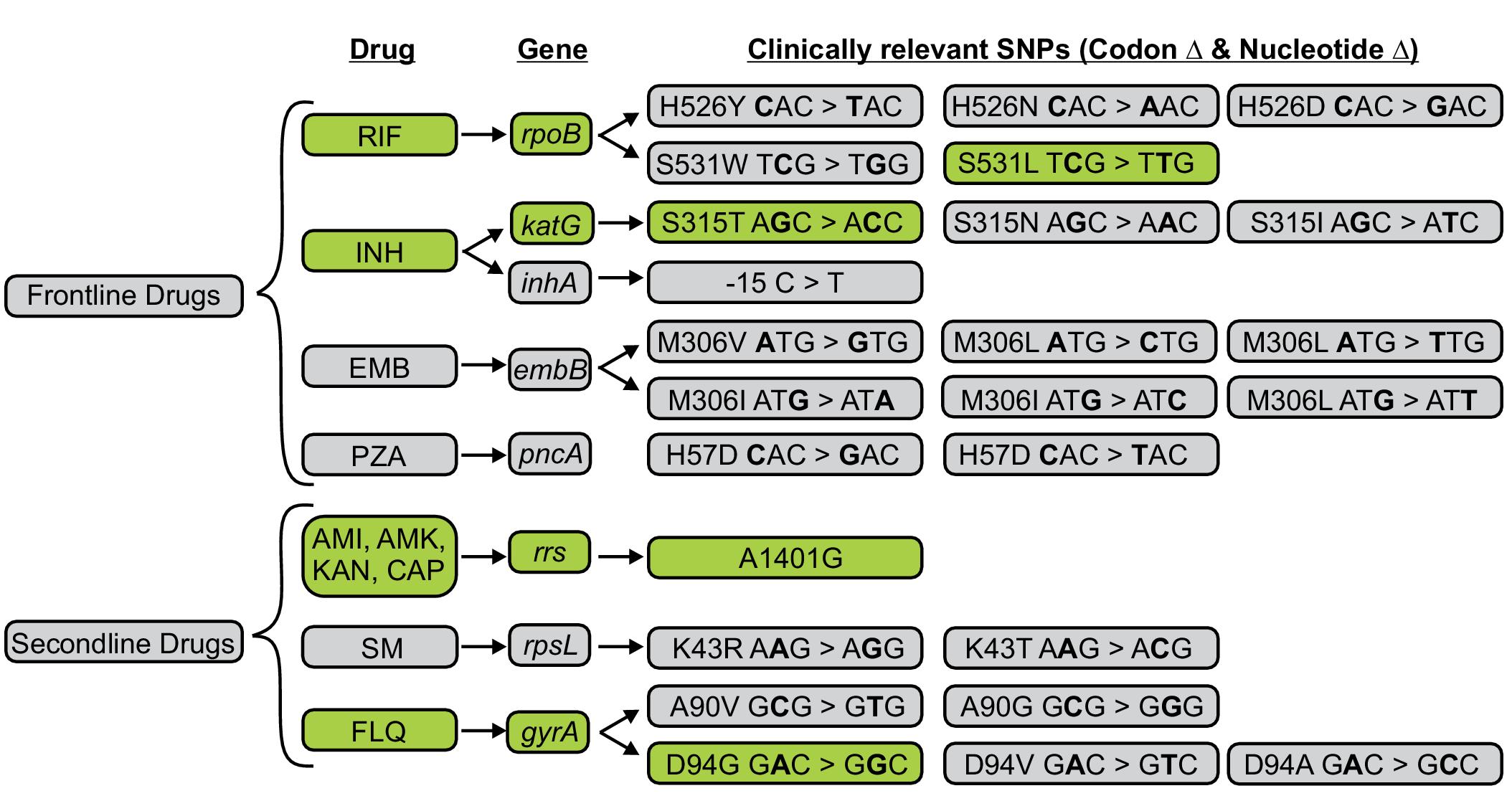


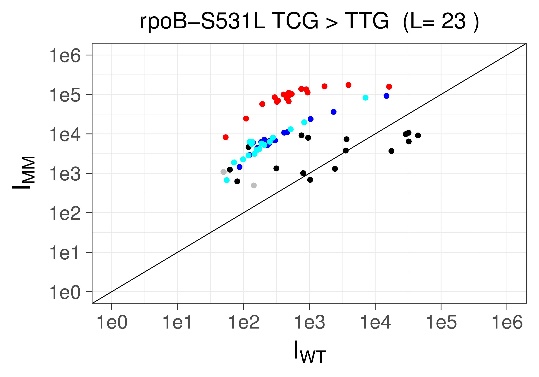

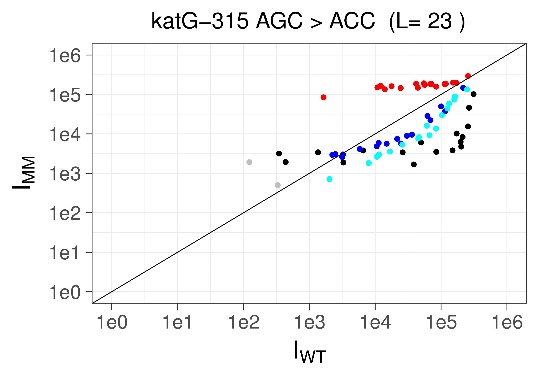

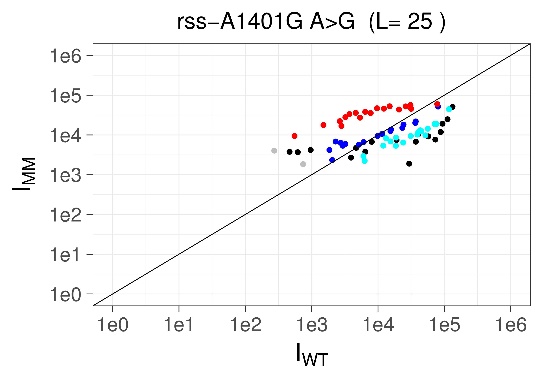

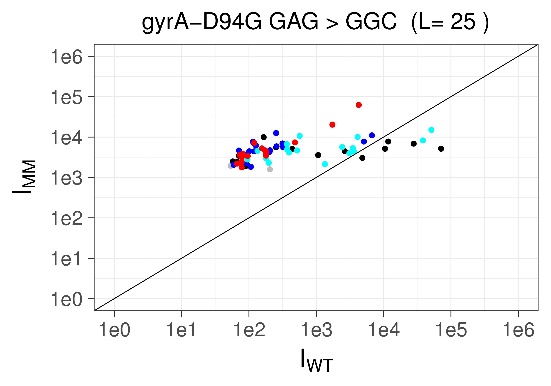


### 08-1186


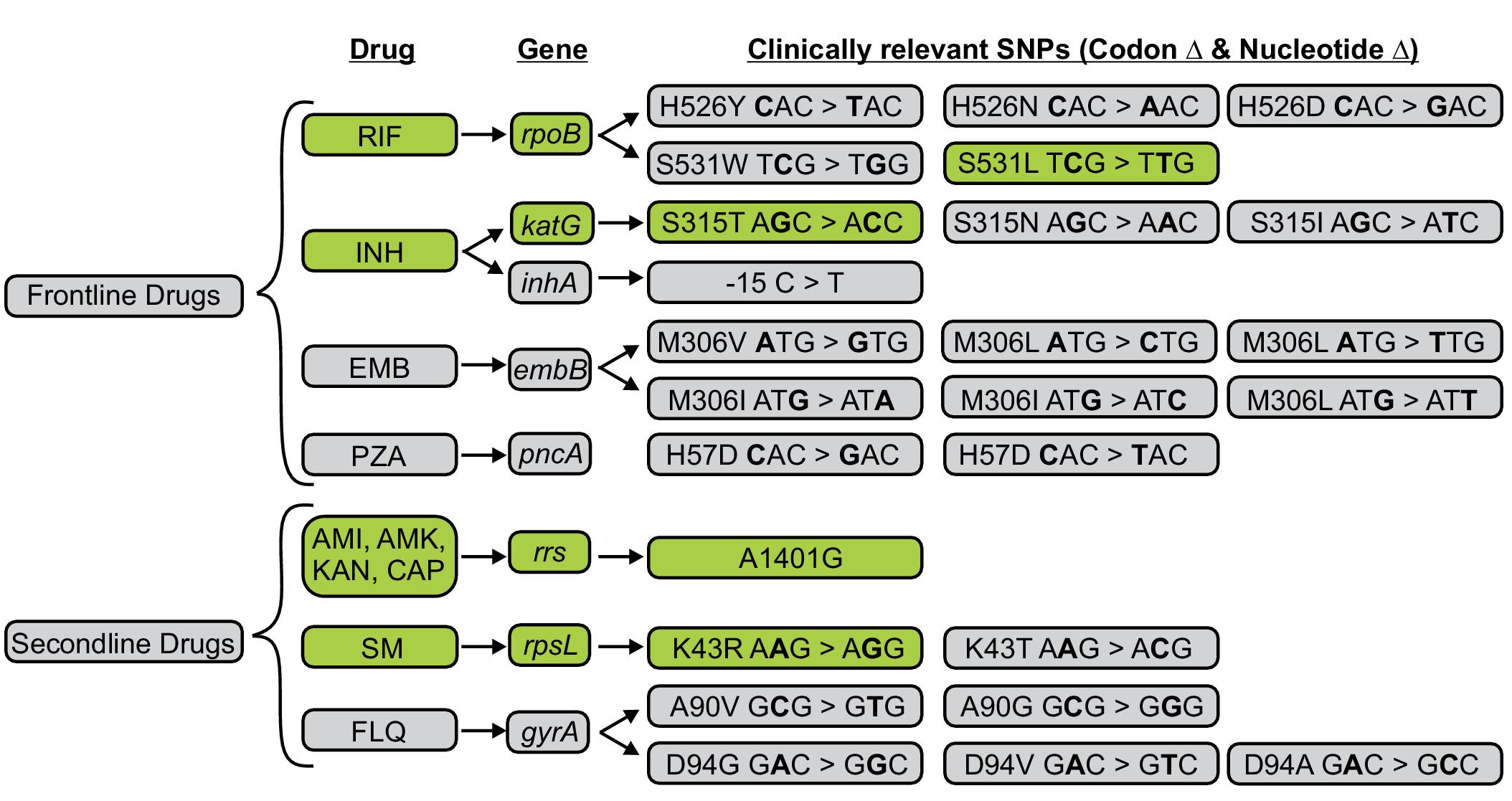


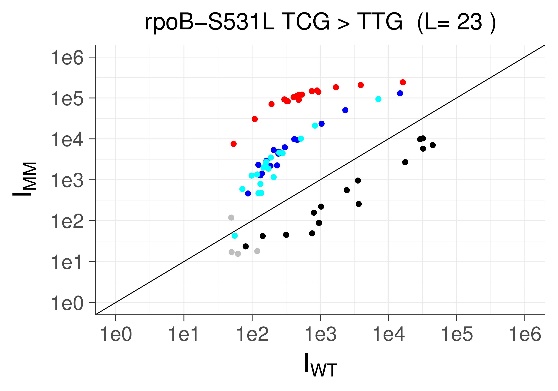

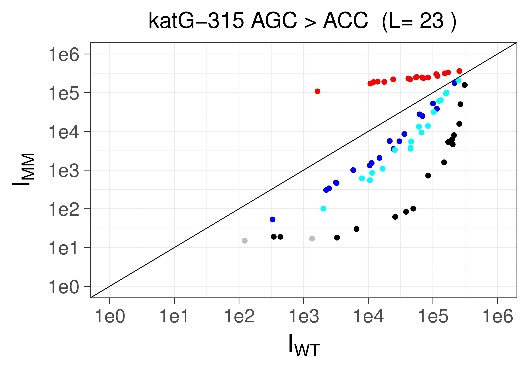

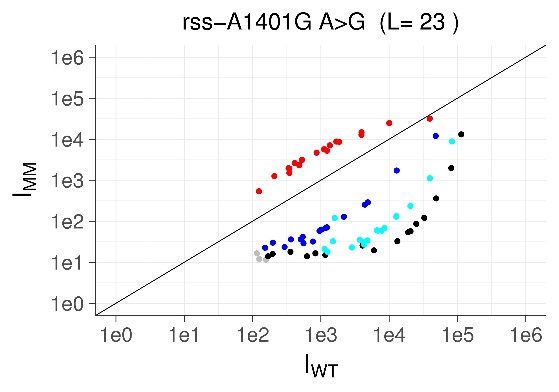

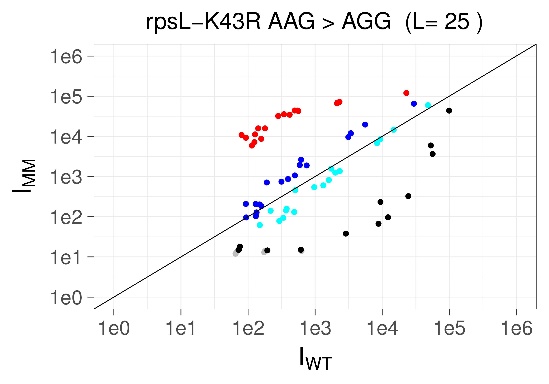


### 03-9532


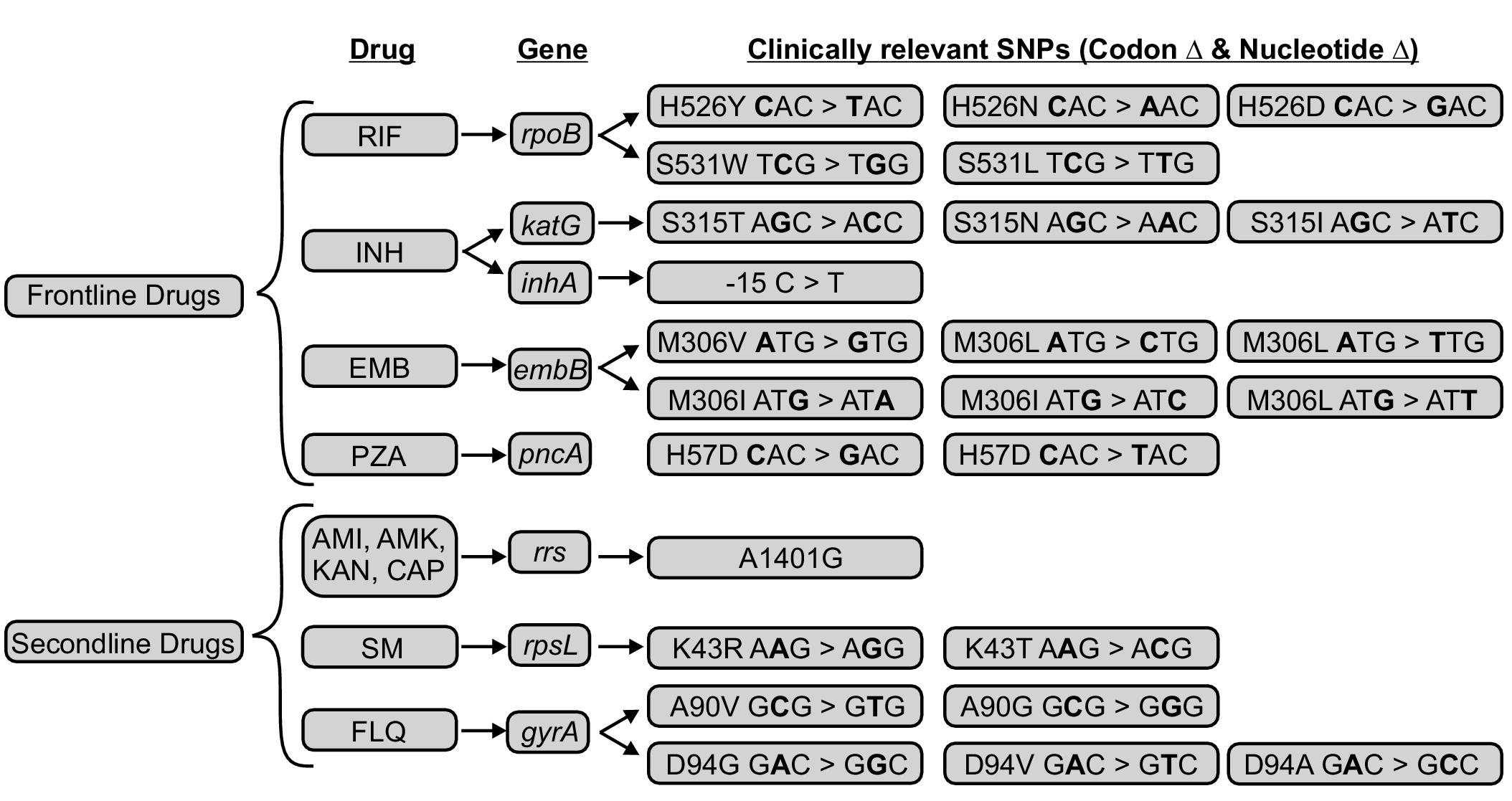


### 03-4850


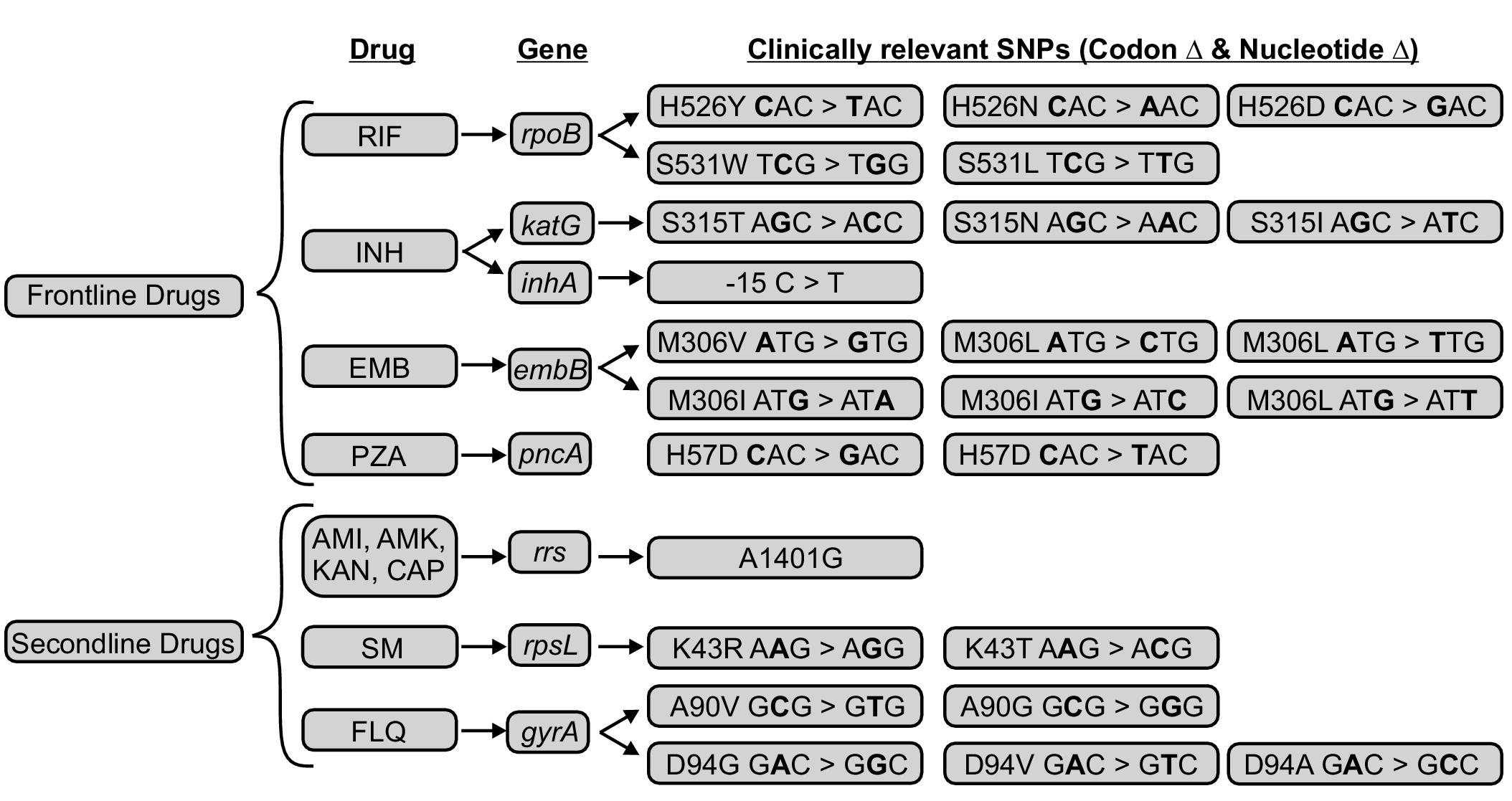


# Strain Typing Schemes & Scatterplots

### 08-1074


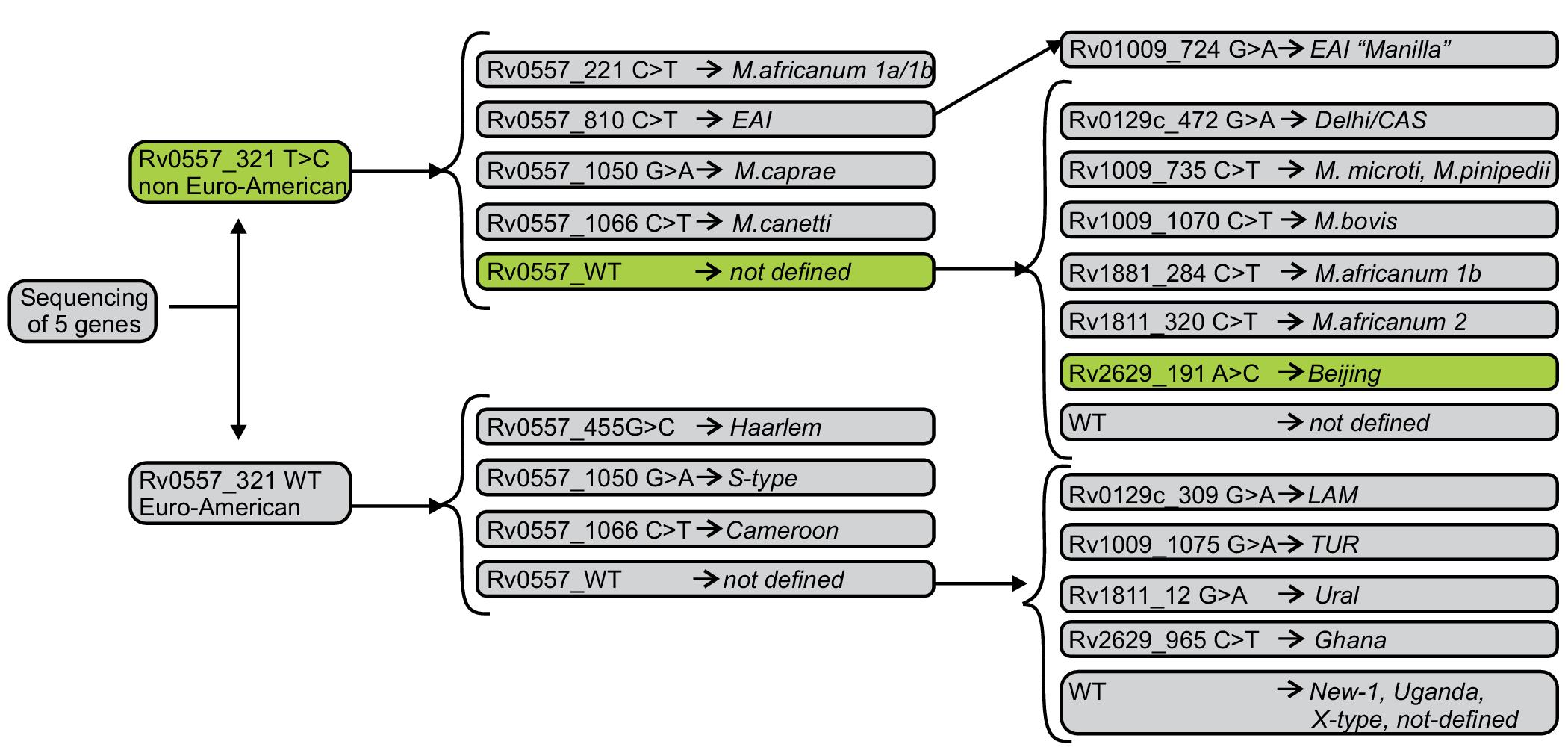


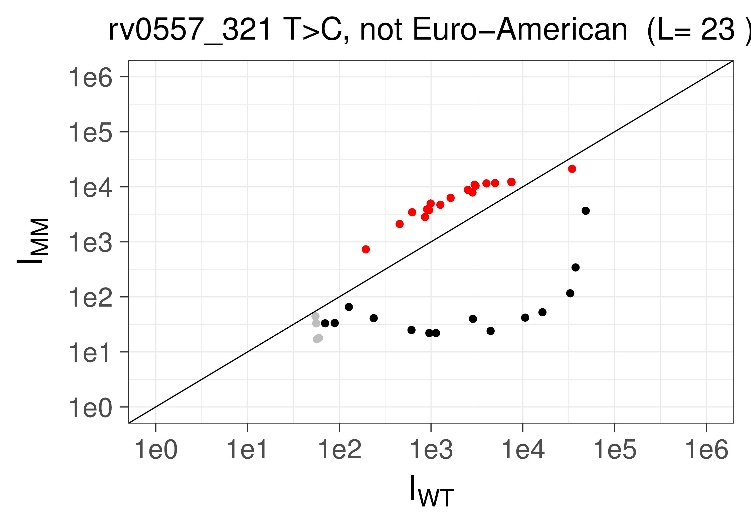

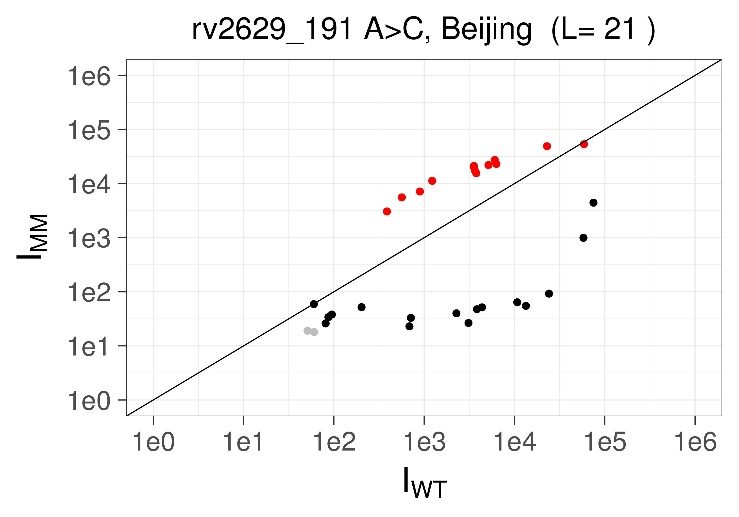


07-2328


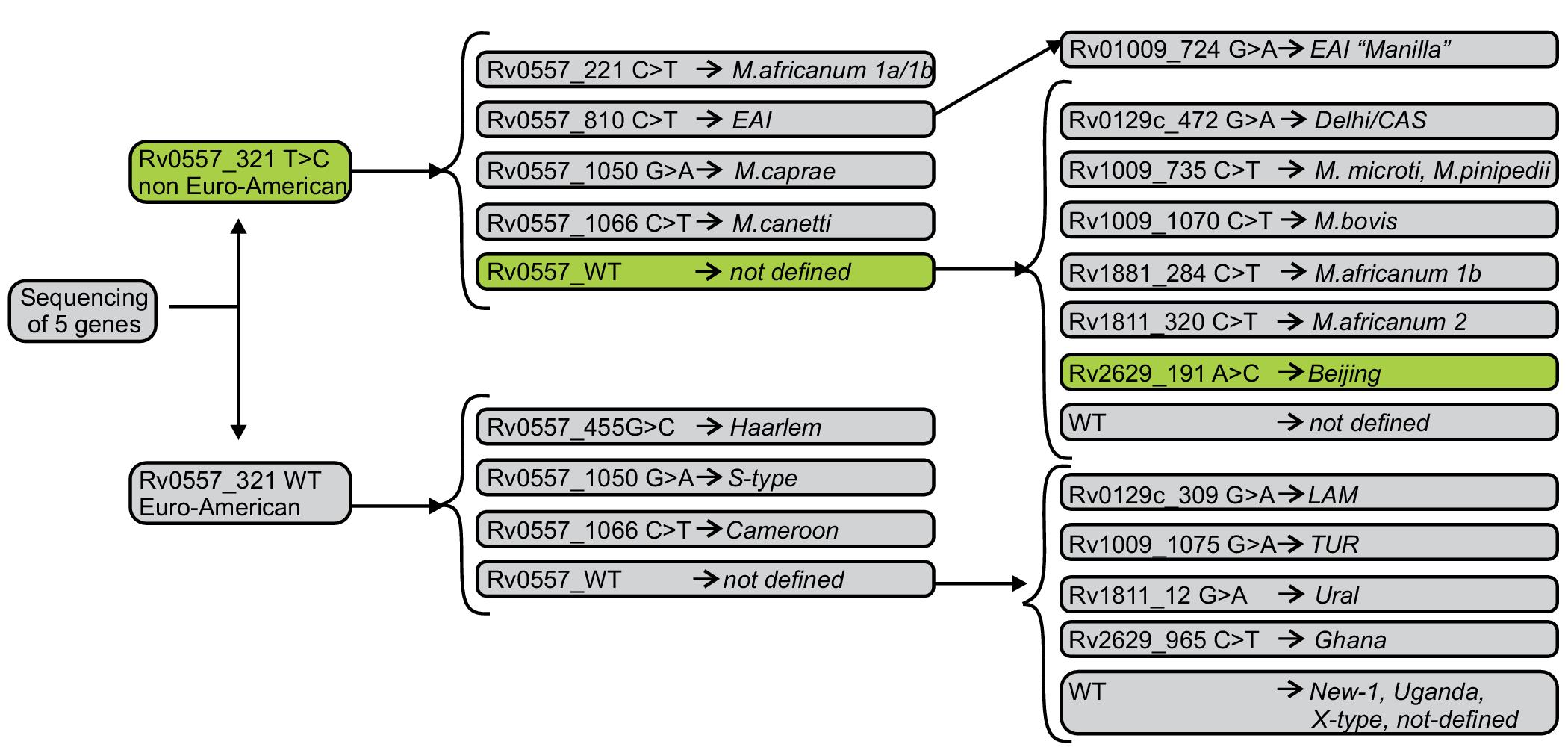


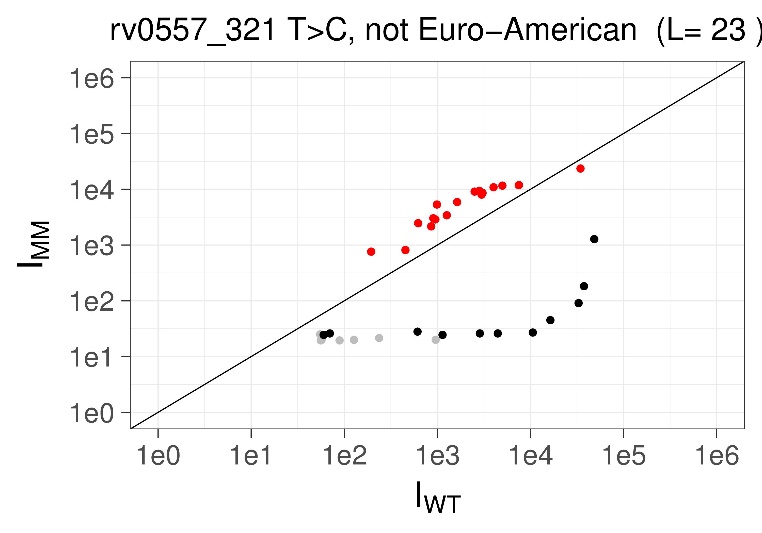

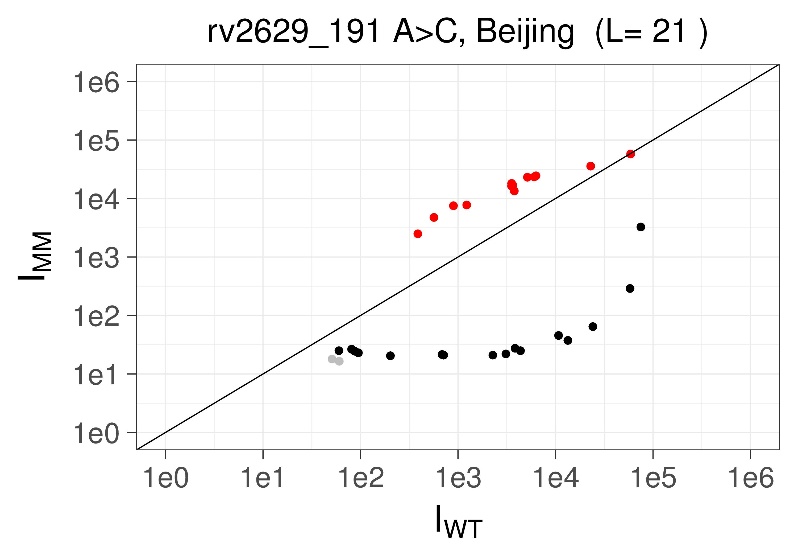


### 09-3657


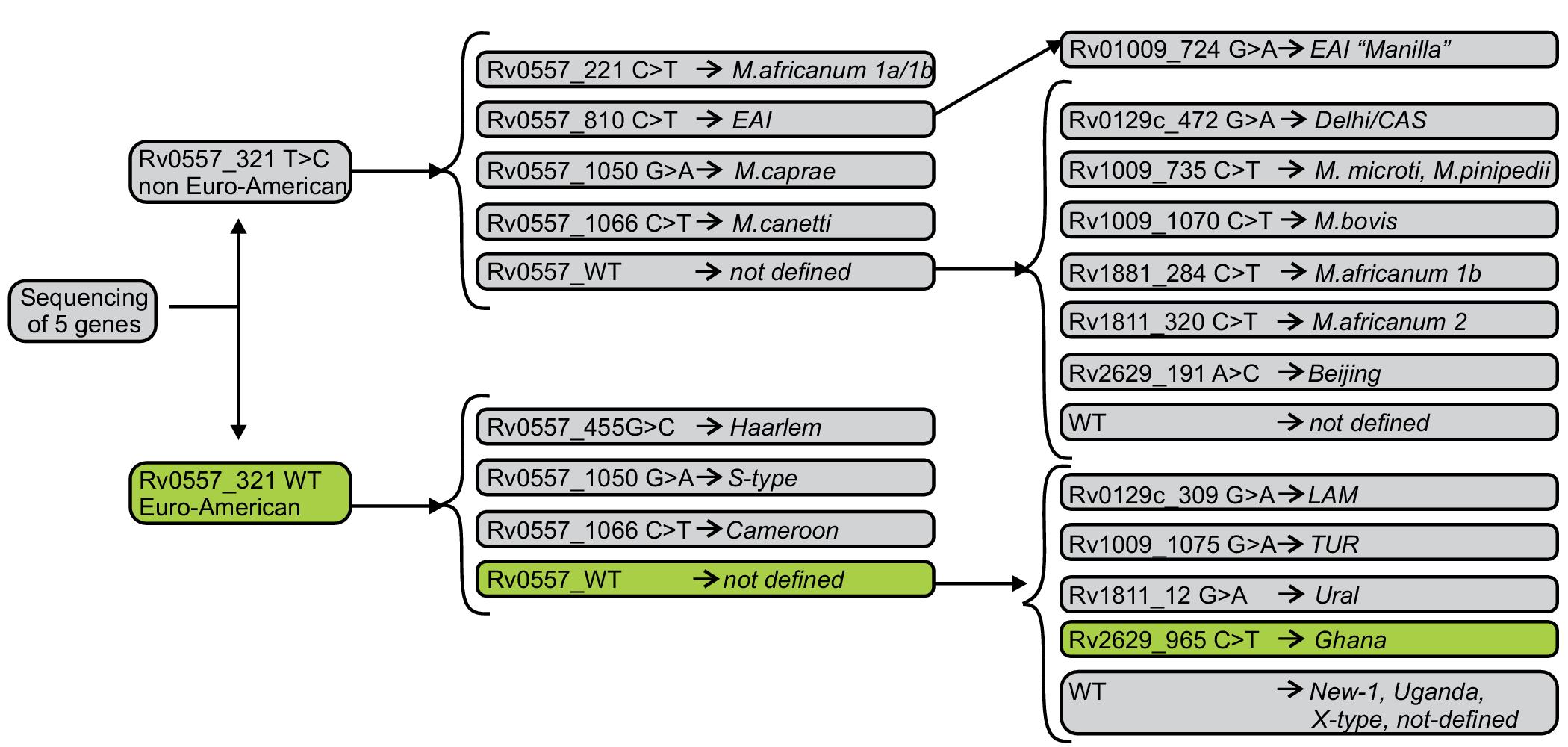


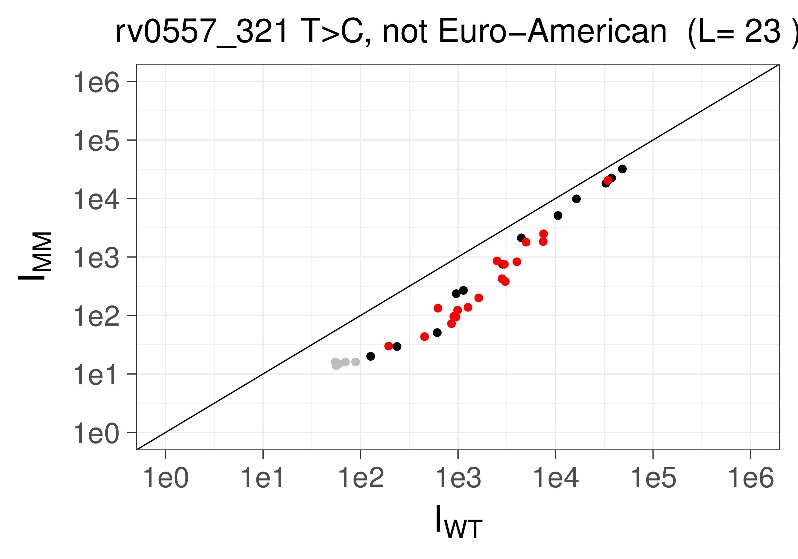

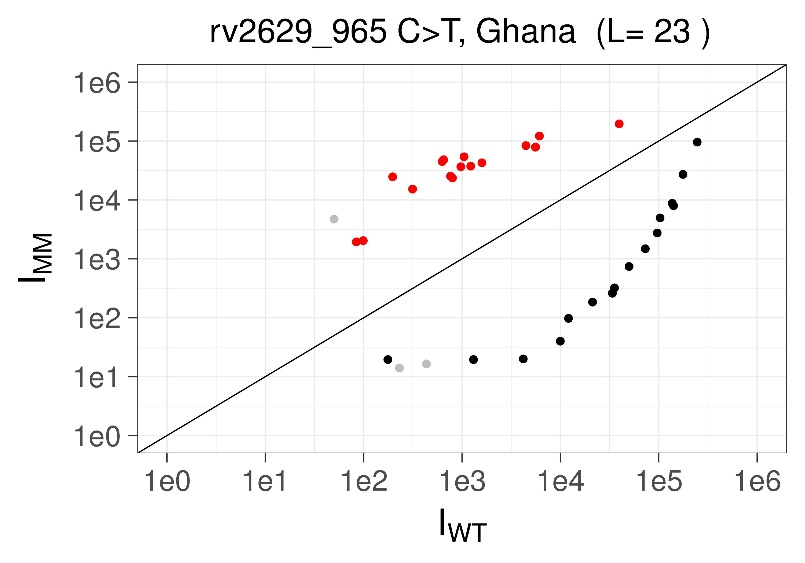


### 07-3082


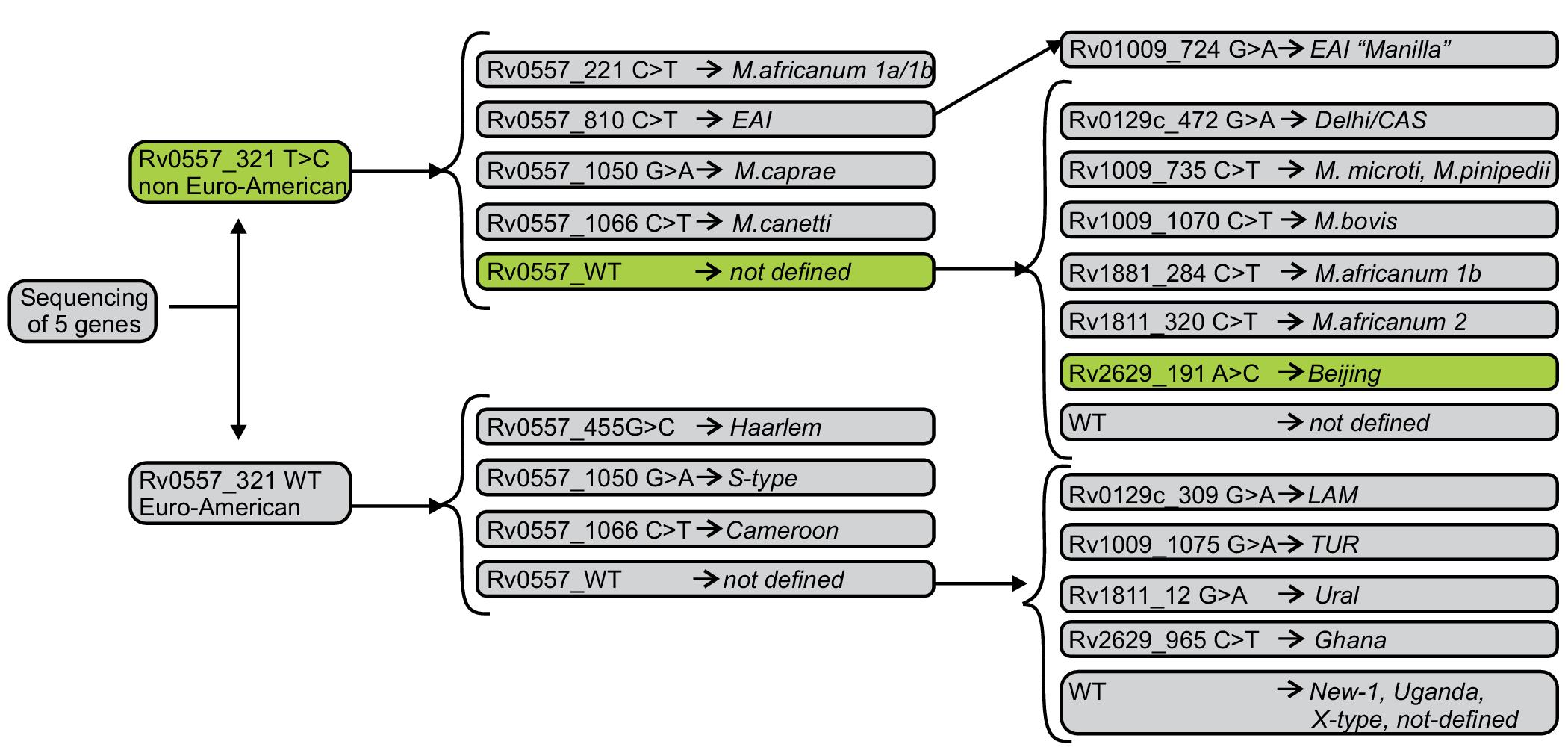


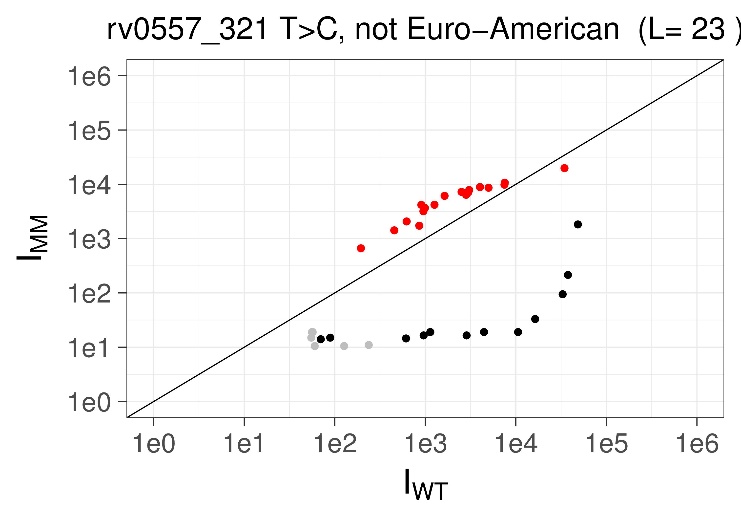

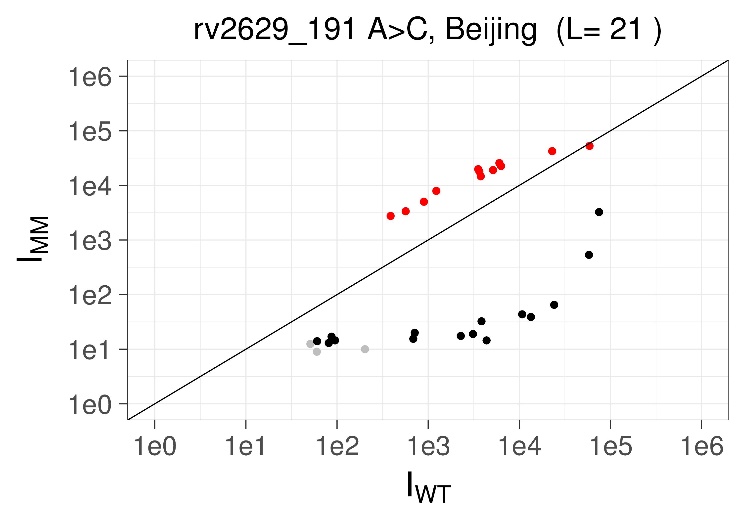


### 07-3216


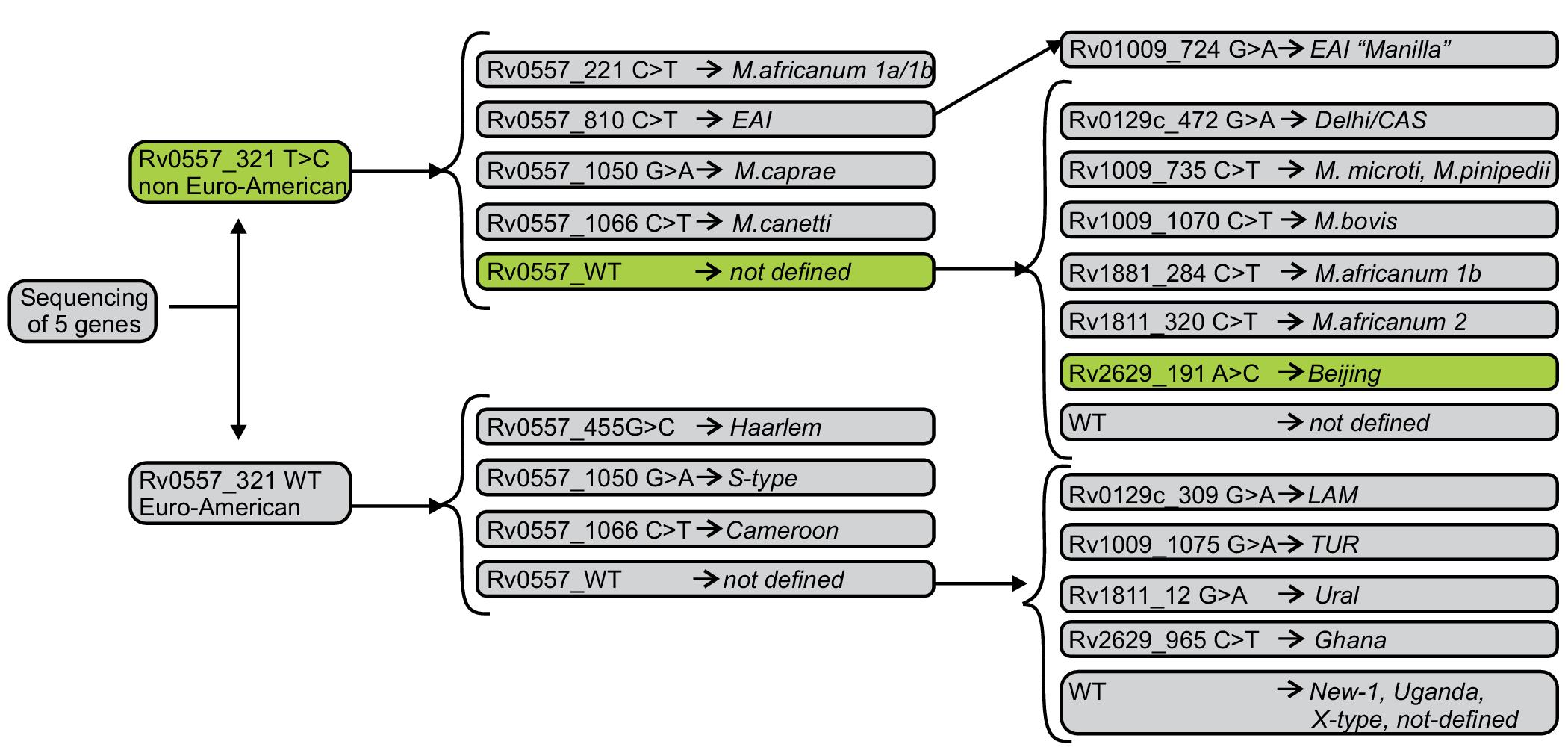


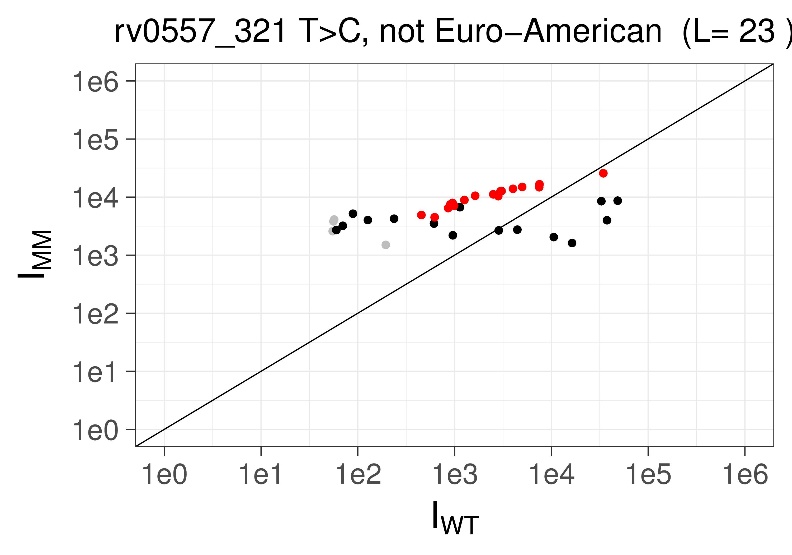

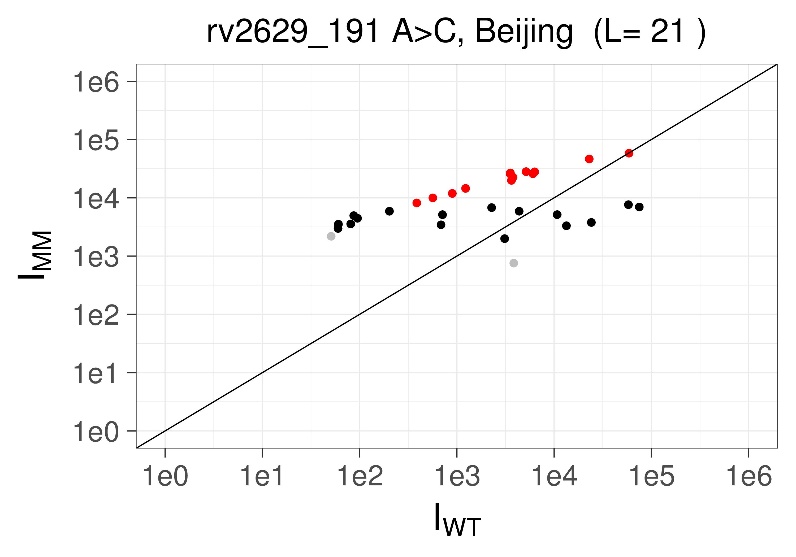


### 08-1186


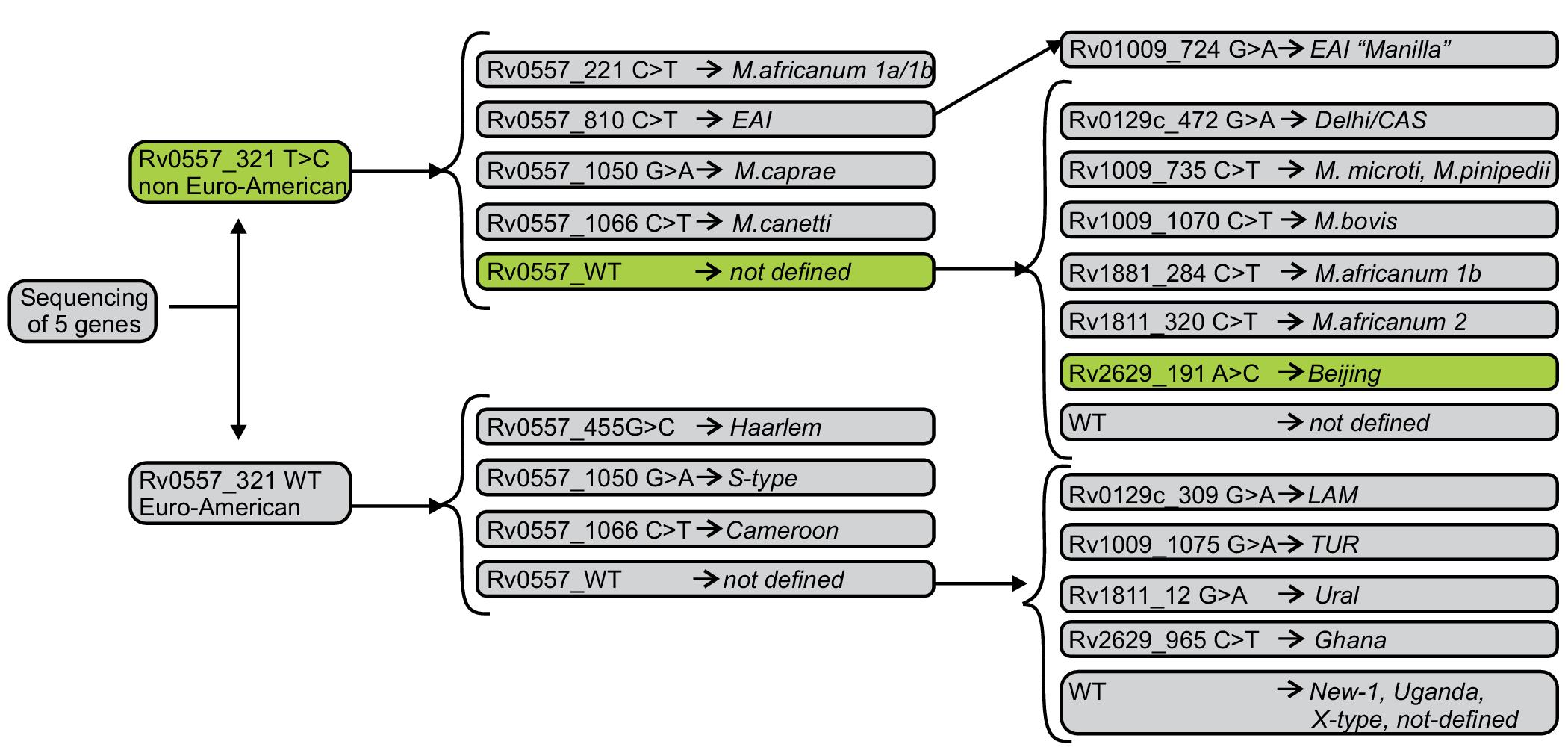


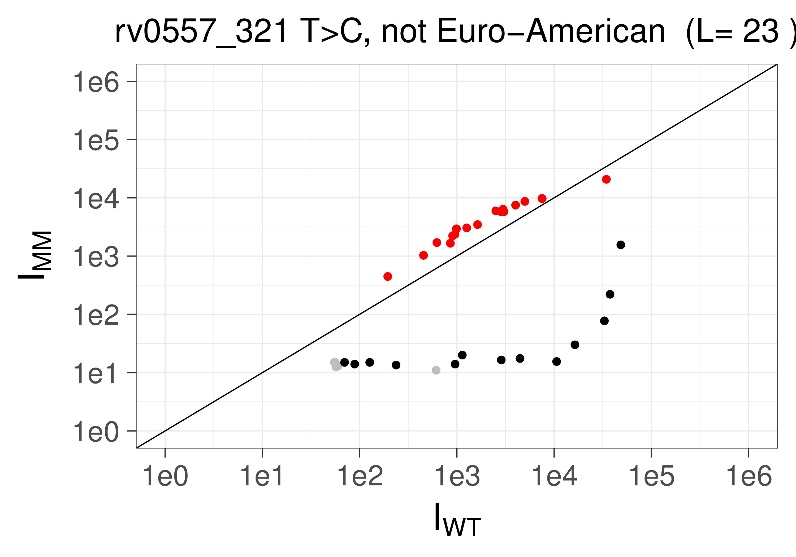

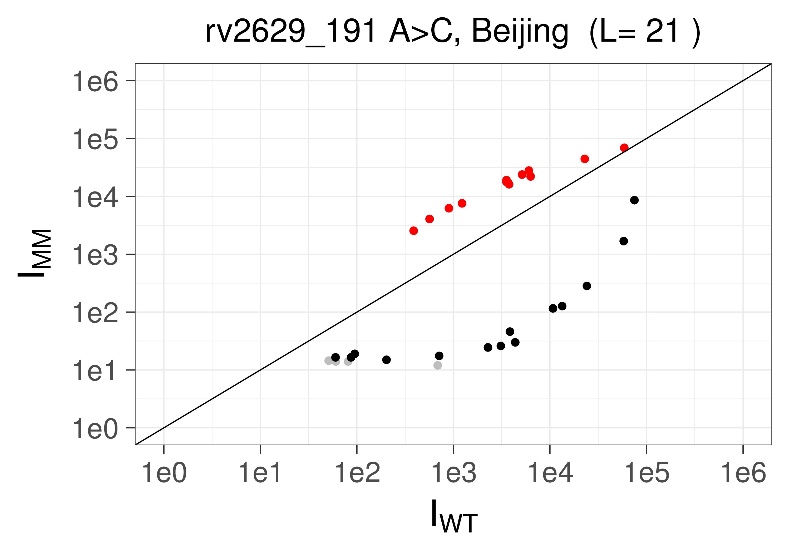


### 03-9532


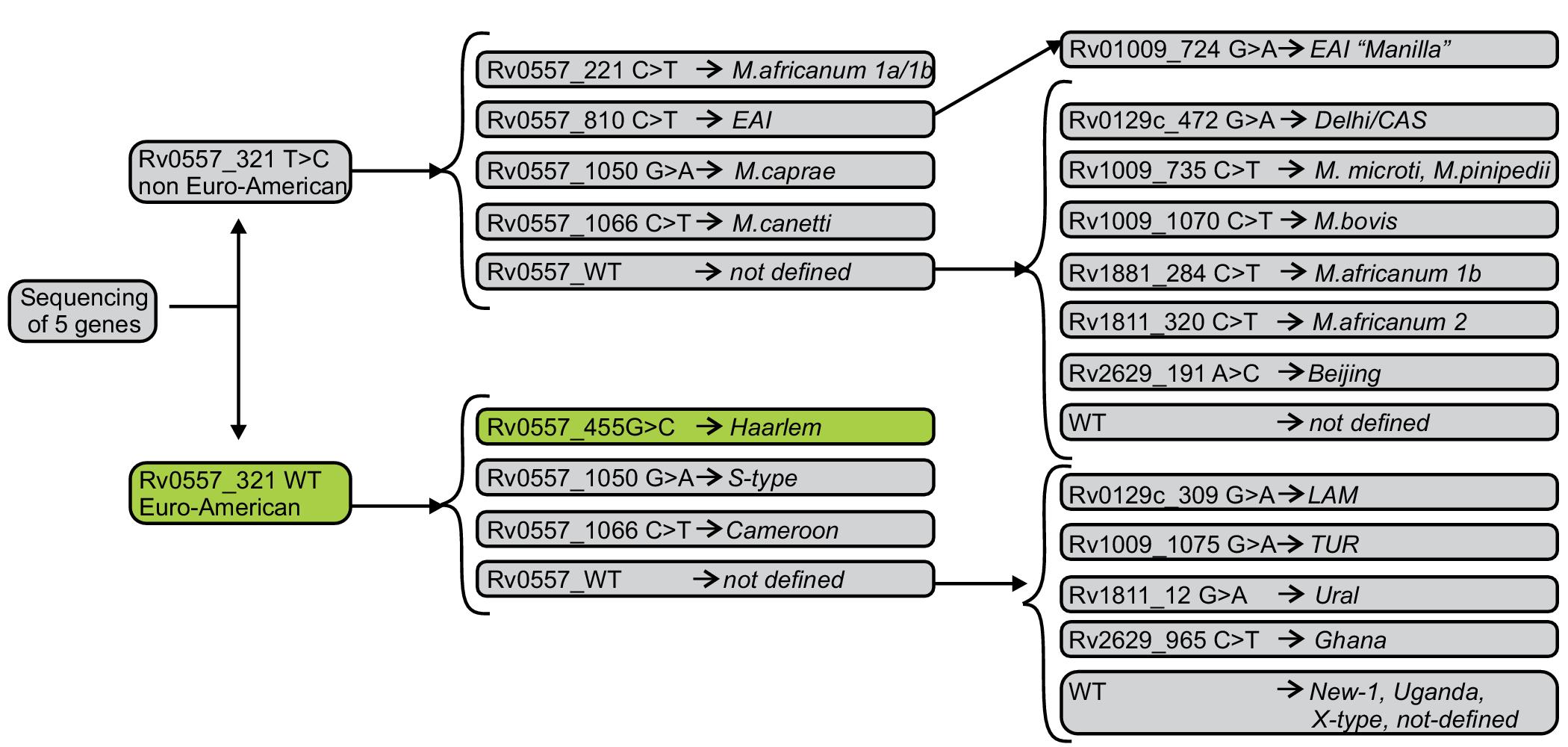


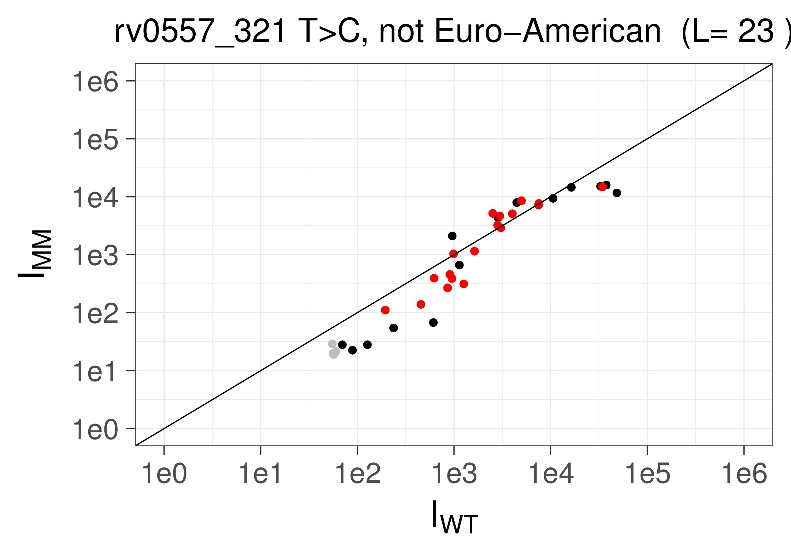

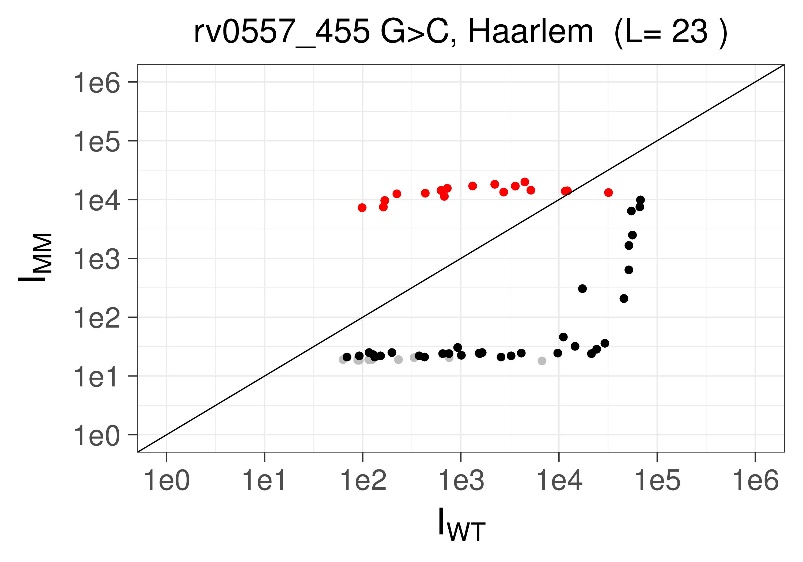


### 03-4850

**
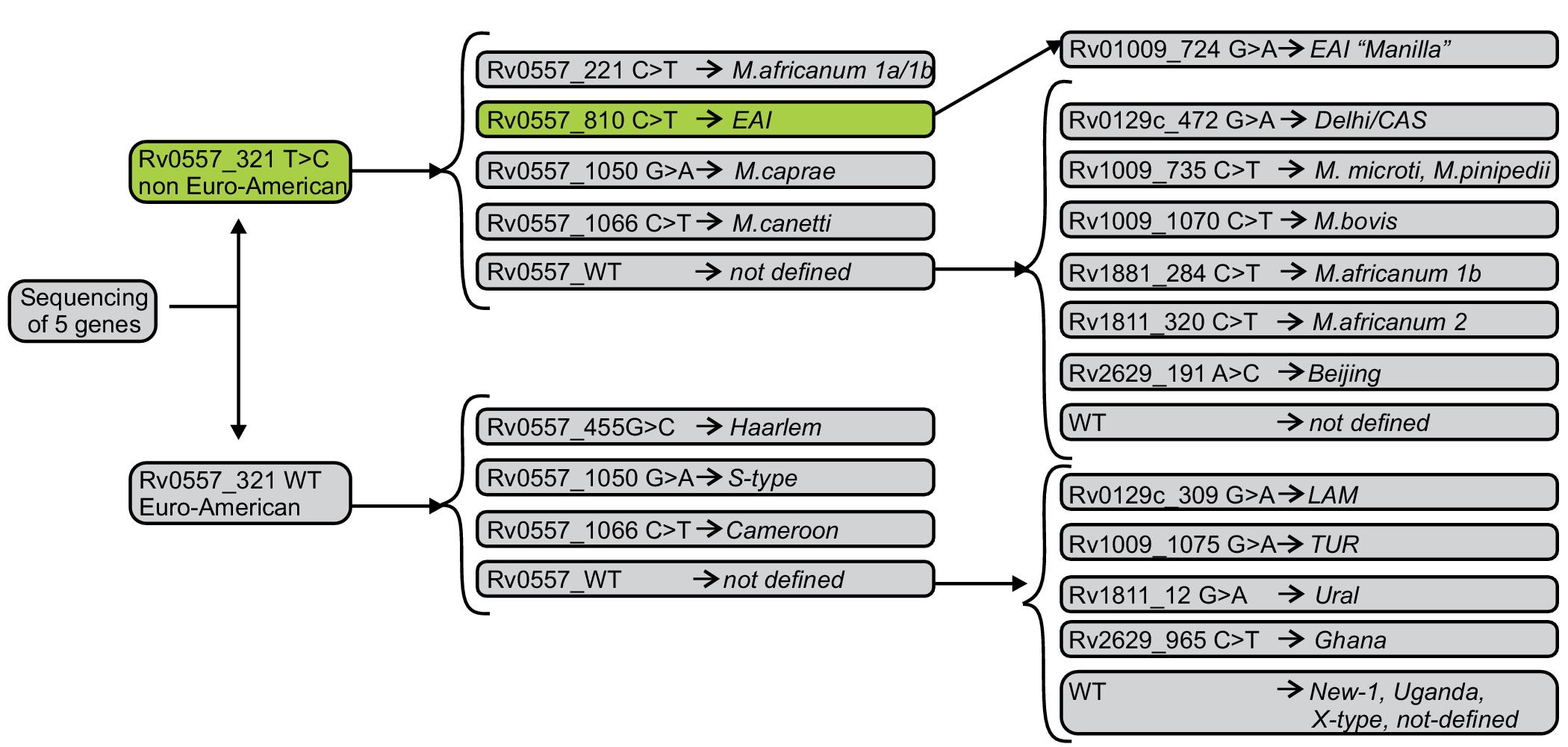
**

**
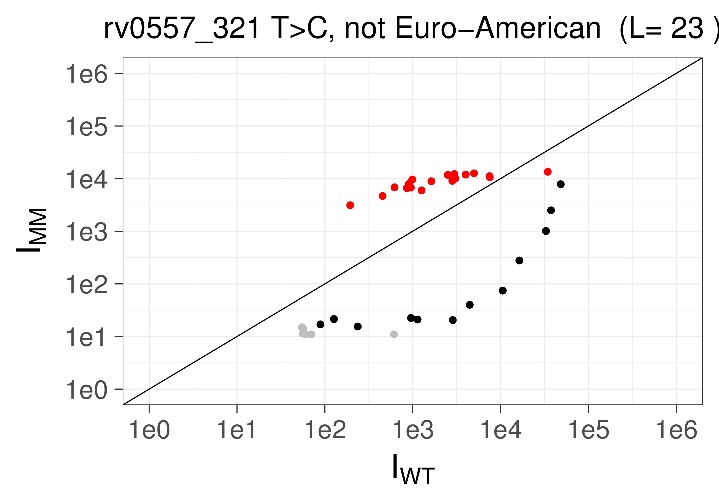

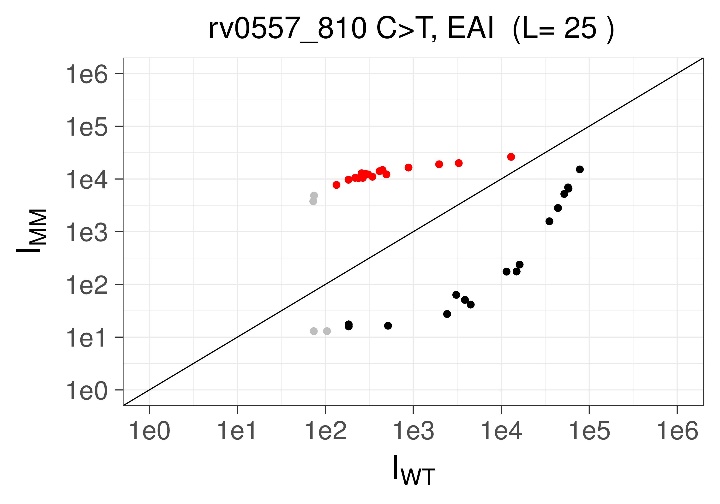
**
